# BMP4 cocktail promotes utricular progenitor reprogramming and vestibular functional recovery in adult mice

**DOI:** 10.1016/j.jare.2025.11.065

**Published:** 2025-11-29

**Authors:** Dan You, Yunzhong Zhang, Kunkun Wang, Luo Guo, Jin Guo, Chenhao Che, Wen Li, Liping Zhao, Huawei Li, Shan Sun

**Affiliations:** ENT Institute and Otorhinolaryngology, Department of Affiliated Eye and ENT Hospital, Key Laboratory of Hearing Medicine of NHFPC, Shanghai Key Laboratory of Gene Editing and Cell Therapy for Rare Diseases, State Key Laboratory of Medical Neurobiology, Fudan University, Shanghai 200031, China

**Keywords:** BMP4 signaling, Progenitor cells, c-Fos, Adult mice, Vestibular function recovery

## Abstract

**Introduction:**

Vestibular hair cells (HCs) are essential for maintaining balance and detecting head movements. In mammals, following vestibular damage HC regeneration derives from epithelial non-hair cells (ENHCs), which possess limited capacity for proliferation and reprogramming.

**Objectives:**

To examine the role of BMP4 in reprogramming utricular HCs following ototoxic injury in postnatal mice.

**Methods:**

The study utilized both wild-type mice and transgenic strains on a C57BL/6J background, including *Notch1*^flox/flox^, *Pou4f3*^+/DTR^, ROSA26^tdTomato^, *Atoh1*-eGFP; *Sox9*-CreER^T2^, and *Fos*-CreER^T2^, to investigate and lineage-trace the reprogramming of new HCs initiated by BMP4 protein. Advanced sequencing techniques, including single-cell RNA sequencing, bulk RNA sequencing, and CUT&Tag sequencing, were employed for transcriptomic and epigenomic analyses. To induce vestibular dysfunction, intraperitoneal injections of IDPN were administered to postnatal day 30 (P30) mice. Vestibular function was assessed through behavioral tests, including vestibulo-ocular reflex, off-vertical axis rotation, and gait analysis, to evaluate the functional outcomes of HC regeneration. For cellular studies, sphere or explant tissues from P2 mice, with gentamicin or small molecule cocktail *in vitro*, were used to evaluate proliferation and differentiation of ENHCs through EdU labelling or tdTomato lineage tracing, histological analyses, immunofluorescence staining, and Western blot analysis.

**Results:**

We found that increased BMP4 expression enhances ENHC reprogramming, accompanied by elevated levels of key HC transcription factors, including *Gfi1*, *Pou4f3*, and *Atoh1*, via c-Fos activation. Moreover, exogenous BMP4 further sensitized ENHCs to Notch inhibition and Wnt pathway activation, thus amplifying the regenerative outcomes. Conversely, inhibition of c-Fos or BMP4 diminished these effects, demonstrating that BMP4 is essential for both Notch inhibition and Wnt activation. Notably, the use of a combination of small molecules targeting these pathways successfully restored vestibular function and promoted HC regeneration in adult mice.

**Conclusion:**

Our findings suggest that BMP4 and its associated signaling pathways represent promising therapeutic targets for the restoration of hearing and balance.

## Highlights

• BMP4 signal is activated post-injury during hair cell regeneration.
• BMP4 signal enhances progenitor reprogramming.
• The role of c-Fos is updated in reprogramming.
• BMP4 analogues offering functional recovery via small molecule cocktails.

## Graphical abstract

**Figure:**
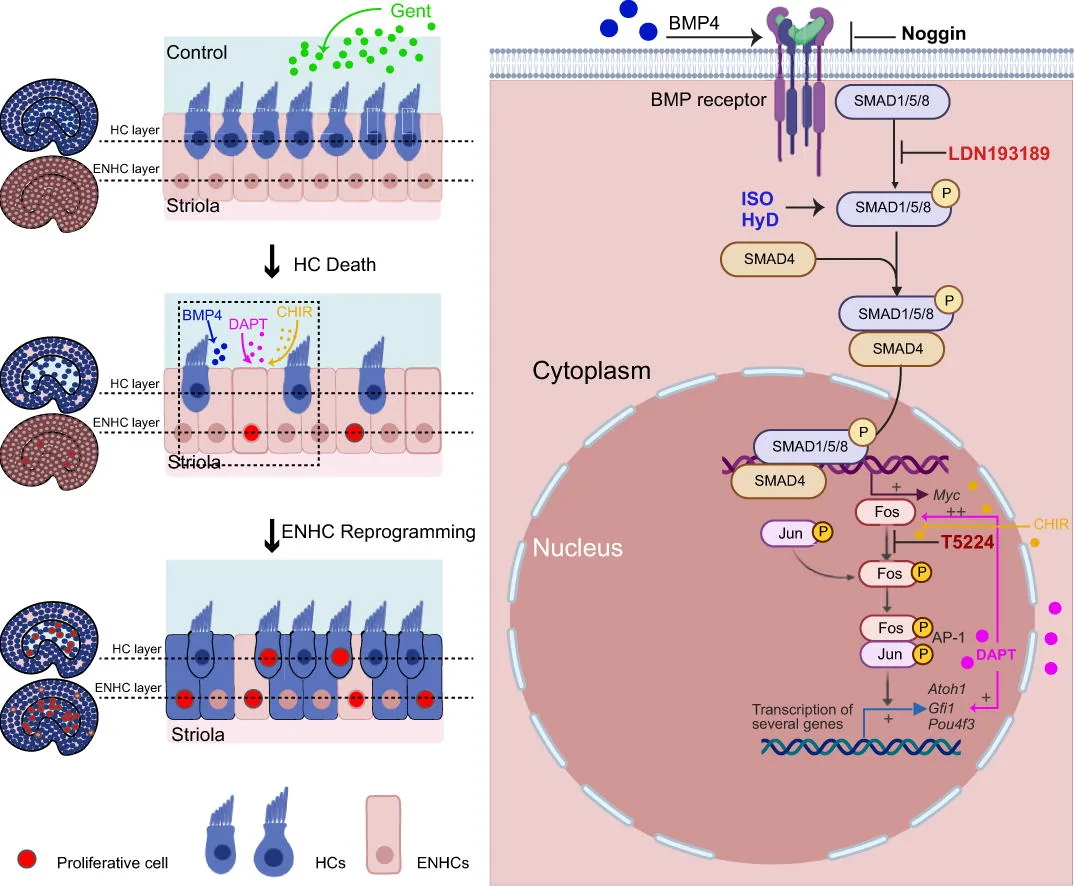

## Introduction

Vestibular disorders can significantly impair everyday functioning and reduce quality of life, and they are often characterized by distressing symptoms such as vertigo, dizziness, and balance loss [1]. These disorders affect one in five adults and their prevalence peaks at 55–64 years of age [2], leading to unsteady gait and increased risk of falls. The vestibular system includes the utricle and saccule maculae along with three semicircular canals, all of which are filled with fluid and lined with sensory epithelium. Hair cells (HCs) housed in the utricular sensory epithelium are critical for providing the brain with information about head movements, especially for detecting linear accelerations and head tilts in the horizontal plane, and this is essential for maintaining balance function and spatial orientation [3].

In mammals, the utricular supporting cells (SCs) or epithelial non-hair cells (ENHCs) [4] display a limited capacity for regeneration, including proliferation and reprogramming to differentiate into HCs following HC loss [[5], [6], [7], [8]], which differs from what is observed in the cochlear sensory epithelium. Regenerated HCs can arise through two primary mechanisms: direct *trans*-differentiation from pluripotent ENHCs and mitotic regeneration from proliferative ENHCs [9].

Several signaling pathways contribute to the reprogramming of ENHCs or differentiation into HCs [[10], [11], [12], [13], [14]]. Notably, Notch signaling is crucial to the process of determining cell fate, with its inhibition leading to the differentiation of cells into HCs [11,15]. Through lateral inhibition, nascent HCs expressing high levels of Atoh1 activate Notch ligands that signal to neighboring progenitors, inducing expression of Hes1 and Hes5, which in turn suppress Atoh1 and direct these cells toward an SC fate. The Wnt signaling network also contributes significantly to both inner ear development and HC regeneration, exhibiting distinct yet complementary roles. The canonical Wnt/β-catenin pathway regulates cell fate, proliferation, and differentiation, in part by activating Sox2 expression [16]. In contrast, the non‑canonical planar cell polarity (Wnt‑PCP) pathway orchestrates cochlear morphogenesis, HC polarization, and the alignment of stereociliary bundles. Increasing evidence indicates that these pathways function in an integrated and cross‑regulated manner, rather than in isolation, to coordinate HC regeneration. Through lateral inhibition, nascent HCs expressing elevated levels of Atoh1 signal to adjacent progenitors via Notch ligands. [15,17,18]. In addition to these signaling cascades, key transcription factors such as *Atoh1*, *Gfi1*, and *Pou4f3* are instrumental in the development of sensory epithelia. The interactions among these factors regulate gene expression and determine cell fate. Previous studies have demonstrated that upregulating the expression of *Gfi1*, *Pou4f3*, and *Atoh1* simultaneously in postnatal SCs *in vivo* can effectively convert these cells into HCs in the cochlea [19,20]. This suggests that increased levels of these transcription factors (collectively referred by the abbreviation GPA) are associated with HC reprogramming or regeneration.

As members of the TGF-β superfamily, bone morphogenetic proteins (BMPs) are essential for various cellular and developmental processes [14,[21], [22], [23], [24]]. BMP4 contributes to the induction and maintenance of pluripotency in mouse embryonic stem cells by promoting their self-renewal [[24], [25], [26]]. During chick inner ear development, BMP4 signaling is an early event, marking the formation of sensory organs and the otic mesenchyme [22], and in mice BMP4 is associated with the early development of the cristae and cochlea [27,28]. The role of BMP4 in HC fate determination has been well-documented [29,30], and recent studies of mice lacking BMP4 in the inner ear have underscored the essential role of BMP4 in the development of the cristae and semicircular canals and in the maturation of the utricle and saccule by modulating gene expression [31]. Despite these findings, there is still much that is not known about the postnatal expression, HC regeneration, and function of BMP4 in the mouse inner ear.

This study examined the role of BMP4 in reprogramming utricular HCs following ototoxic injury in postnatal mice. Our findings demonstrate that exogenous BMP4 plays a critical role in enhancing reprogramming by increasing the responsiveness of ENHCs to multiple signaling pathways, which is mediated through the upregulation of key GPA transcription factors. Notably, we identified several BMP4 target genes, including c-Fos, a transcription factor predominantly active in ENHCs within the striolar zones. BMP4 significantly elevated c-Fos activation, which in turn increased the expression of downstream target genes such as *Myc*, *Smad1*, and *Ids*. Furthermore, the combined application of BMP4 and Notch inhibition significantly boosted the expression of *Atoh1*, *Gfi1*, *Pou4f3*, and *Myo6*, all of which are critical for HC *trans*-differentiation. Additionally, activating BMP4 signaling through a small molecule cocktail in the vestibular system of adult mice promoted HC regeneration and partially restored vestibular functions. Our study suggests that BMP4 can promote inner ear HC regeneration by enhancing ENHC reprogramming through c-Fos activation in both neonatal and adult damaged utricles, further opening avenues for the development of targeted therapies that harness the potential of cell reprogramming to facilitate tissue regeneration and repair.

## Methods and materials

### Mice

Wild-type C57BL/6J mice were sourced from Jiesijie Laboratory Animal and Shanghai Lingchang Biotech Co. Ltd. The following transgenic strains in C57BL/6J background were used: Notch1flox/flox (#007181), Pou4f3+/DTR (#028673) [32], and ROSA26tdTomato (Ai14, #007908) [33] from Jackson Laboratory; Atoh1-eGFP from Jane Johnson (University of Texas Southwestern Medical Center); Sox9-CreERT2 from the Shanghai Academy of Life Sciences; and Fos-CreERT2 (NM-KI-200110) from Shanghai Model Organisms Center, Inc.

All experiments used mice of both sexes and were performed under protocols approved by the Animal Care and Use Committee of Fudan University (202412007Z) and in accordance with NIH guidelines. Tamoxifen (Sigma-Aldrich, prepared in a corn oil vehicle) was administered intraperitoneally (0.20 mg/g body weight) at postnatal day 1 (P1) or P2 for Cre activation. HC ablation in Pou4f3+/DTR mice was achieved through intramuscular Diphtheria toxin injection (List Biological Laboratories, 25 ng/g) at P30 with a second dose after two days. P30 represents the onset of early adulthood in mice and is widely recognized as a standard time point for vestibular research. At this age, vestibular hair cells are fully differentiated and functionally mature, allowing the use of a stable, adult model that minimizes developmental variability while avoiding potential confounding factors associated with age-related cellular degeneration [[34], [35], [36]].

### Genotyping and real-time polymerase chain reaction (RT-PCR)

For genotyping, genomic DNA was isolated from 0.5 cm tail samples with a Direct PCR Lysis Reagent containing proteinase K (20 mg/mL). The lysis protocol included sequential one-hour incubations at 98 °C and 85 °C, followed by centrifugation to remove debris. The resulting supernatant was used as the template for PCR, with primers listed in Supplementary Table 1.

For expression analysis, total RNA was extracted using RNAiso Plus and reverse-transcribed into cDNA via the PrimeScript II 1st Strand cDNA Synthesis Kit. Quantitative PCR (qPCR) was then performed on a 7500HT Fast Real-Time PCR System using SYBR Premix Ex Taq II. All reactions were run in triplicate, and relative gene expression was calculated using the ΔΔCT method with GAPDH as the reference gene. The primers for qPCR are provided in Supplementary Table 2.

### Tissue collection and culture

Inner ears were harvested from 6 to 8 embryos of timed-pregnant females or 10–15 postnatal animals of either sex. For culture experiments, utricles from embryonic day 16 (E16), P2, and P30 mice were dissected from the temporal bone, with tectorial membrane and otoconia removed under sterile conditions. The specimens were attached to CellTak −coated (BD Biosciences, # 354240) coverslips in 4-well Petri dishes.

Cultures were maintained in serum-free medium containing DMEM/F12 (1:1; Invitrogen), N2 (1:100), B27 (1:50), and ampicillin (50 ng/ml) at 37 °C with 5 % CO2. HC damage was induced using gentamicin sulfate (2 mM, Sigma) for 24 h, followed by 5-day treatment with various reagents (Supplemental Table 3). EdU (10 μM) was included throughout the culture period. For fate tracing studies, 4-hydroxy-tamoxifen was added to activate Cre recombinase in transgenic mice. Culture medium was refreshed daily.

### Sphere-forming culture

For sphere culture, single cells were obtained by trypsin digestion as described [37,38]. The cell suspension was then mechanically dissociated using plastic pipette tips (epTIPS Filter, 20–300 μL; Eppendorf) and subsequently filtered through a 40-μm cell strainer (Greiner Bio-One) to ensure a uniform single-cell population.

### Dose-response curves

Dose-response curves for various reagents were generated using the following procedure. A total of 100 mice were assigned to the different groups, and their isolated utricles were subjected to *in vitro* experiments to assess the damage caused by gentamicin. The utricles were exposed to cocktails of small molecules at specified concentrations as follows: BMP4 (5 ng/ml to 200 ng/ml), ISO (7.5 µM to 60 µM), Hyd (1 µM to 20 µM), T-5224 (3 µM to 20 µM), and LDN-193189 (0.1 µM to 1 µM) (Fig. S1). For each reagent, 4–5 doses were tested in each experiment with 6–8 utricles used per dose. The respective protein or chemical was added to the culture medium. The IC50 value was calculated for inhibitors, while the EC50 value was calculated for agonists. All data points and curve fittings were generated using GraphPad Prism 6.0 software.

### Drug delivery

To establish the model of vestibular HC injury in adult mice, 5 µl/g IDPN was injected intraperitoneally into P30 mice. The thermo-reversible triblock copolymer P407 was used as the drug delivery vehicle for local injection into the middle and inner ears. This has been shown to be an effective and safe delivery system to achieve sustained release of drugs in the inner ear [39,40]. P407 hydrogel (30 %, Sigma #16758-250G) was prepared in PBS, stored overnight at 4℃ for complete hydration, then mixed with drugs at specified concentrations. Four-week-old C57BL/6 mice were anesthetized with ketamine (100 mg/kg) and xylazine (25 mg/kg). Using a post-auricular approach, the otic bulla was exposed and carefully opened to visualize the round window niche, identified based on its anatomical position relative to the temporal bone and facial nerve. A glass micropipette with a 25-μm tip diameter, controlled by a micromanipulator (UMP3 UltraMicroPump, WPI), was used to precisely deliver 10 μL of drug-loaded P407 gel onto the surface of the round window. Following the procedure, the incision was sutured and treated with levofloxacin to prevent infection. The surgery was performed exclusively on the left ear of each mouse. To monitor cell proliferation, EdU (5 mg/mL, 50 μg/g body weight) was administered daily via intraperitoneal injection from P37 to P51.

### Vestibular function tests

Vestibular function was assessed four weeks after the injection of small-molecule compounds using a binocular video-oculography (VOG)-based vestibular function test (VFT) system, developed by Prof. Fangyi Chen’s team at the Southern University of Science and Technology. To assess semicircular canal and macular organ function, HAVOR (horizontal angular vestibulo-ocular reflex) and OVAR (off-vertical axis rotation) tests were performed. Mice were secured on a weight-adjusted rotating platform using a noninvasive immobilization device as previously described [41,42]. Under infrared illumination, eye movements were synchronously recorded via side camera using mirror images. For HAVOR testing, rotational stimuli were delivered at frequencies of 0.25 Hz, 0.5 Hz, and 1 Hz for minimum durations of 120 s, 100 s, and 80 s respectively to obtain sufficient video data. For OVAR testing, angular velocities of 30, 50, and 80 degrees per second were applied. The exported eye position data were processed using Fourier transformation in MATLAB 2020 to extract eye movement amplitude. Gain values were then calculated as the ratio of response amplitude to stimulus amplitude.

Ocular vestibular evoked myogenic potential Vestibular Evoked Myogenic Potential (oVEMP) recordings were additionally utilized to evaluate vestibular functional recovery. Following anesthesia, mice were secured in a custom holder with ocular stabilization. EMG signals were recorded from the contralateral inferior oblique muscle following acoustic stimulation (4 ms clicks, 5 Hz) via insert earphones. Signals were amplified 100,000 × and band-pass filtered (30–3000 Hz). Thresholds were determined by decreasing stimulus intensity from 110 dB SPL in 10 dB steps and confirmed with duplicate recordings.

The automated Noldus CatWalk XT 2020 system, which acquires, compresses, stores, and analyzes the pass processes of animals crossing the walkway, was used to evaluate the gait and movement of rodents [[43], [44], [45]]. Animals walked across an opaque walkway with a glass floor in a darkened room. The system uses a white fluorescent tube that emits light into the glass floor's edge. Due to total internal reflection, light only exits where paws contact the glass, illuminating the contact areas. Infrared beams detect the mouse's entry and control data acquisition. The floor is monitored by a video camera with wide-angle lens via mirror. During the initial pass, bright areas in the frames are automatically detected, labeled, and indexed. In the subsequent pass, these spots are classified as the right or left forepaw, hind paw, nose, abdomen, tail, or artifacts.

The system records spot positions, sizes, contents, and categories. Mean pressure density is calculated by averaging pressure readings across all footprints during set period/steps using the software given by the equipment [44]. Post-injury values were compared with post-repair values for different drugs using unpaired Student's t-tests, with *p* < 0.05 considered statistically significant.

### RNA sequencing and analysis

RNA extraction was performed on pooled utricles (n = 12) using AllPrep DNA/RNA/Protein Mini kit (QIAGEN), maintaining three biological replicates for each genotype. Library construction utilized NEBNext Ultra kit with indexed adaptors. The workflow included size selection, enzymatic treatment, and high-fidelity PCR amplification. Library quality was verified by Bioanalyzer analysis prior to cluster generation and paired-end sequencing on Novaseq6000 platform.

Computational analysis involved read preprocessing with TrimGalore (v0.6.10), genomic alignment to mm10 (Ensembl release-110) reference using HISAT2 (v2.2.1), and transcript quantification by featureCounts (v2.0.6). Gene expression changes were identified through DESeq2 (v1.34.0) analysis, with significance criteria of adjusted *p* < 0.05 and |log2FC|>0.25.

### CUT&Tag sequencing and data analysis

CUT&Tag was performed using Hieff NGS Kit (Yeasen, 12598ES12). Utricle explants were dissociated using Accutase (Thermo Fisher Scientific) at 37 °C. Lysates (10,000 cells) were sequentially incubated with: concanavalin A-coated beads (10 min, RT), c-Fos primary antibody (1:50, Abcam ab25949) or IgG control (Millipore 12–370) overnight at 4 °C, secondary antibody (1:100, Millipore AP132) for 2 h, and pA/G-Tn5 complex for 1 h. DNA was purified with Yeasen kit (12601ES03) including E. coli spike-in DNA. Libraries were generated using Hieff NGS Tagment Index Kit (Yeasen, 12610ES96), validated on Agilent 4200 TapeStation, and sequenced (150 bp paired-end) on NovaSeq 6000. Analysis was performed as previously described [46]. Differential peaks were identified using edgeR, and genomic data were visualized in Integrative Genomics Viewer (v2.16).

### Isolation of utricular cells for scRNA-seq analysis

Utricle sensory epithelia from E14 and P2 mice were isolated using thermolysin treatment and dissociated with Accutase at 37 °C. After centrifugation and filtration through a 40-µm strainer, cell viability was assessed using trypan blue. Libraries were generated using 10X Genomics Chromium platform with Next GEM Single Cell 3′ Kit v3.1, targeting 20,000 cells per sample.

Sequencing data were processed by aligning raw fastq files to mouse genome GRCm39 using Cell Ranger 3.1.0. Analysis employed Seurat (v4.1.1) with SCTransform normalization. Quality control removed cells based on read counts and mitochondrial content, followed by dimensionality reduction via PCA and UMAP. Cell clustering was performed using FindNeighbors and FindClusters functions. Cell type annotation utilized Garnett (v0.2.8) with established markers, and cross-timepoint integration was achieved using Harmony.

### Western Blotting, immunohistochemistry and analysis

Protein analyses were performed by extracting utricle proteins using proteinase inhibitor-containing lysis buffer, separating via SDS-PAGE, and transferring to PVDF membranes (Immobilon-P; Millipore). After blocking with 5 % milk in TBS/0.1 % Tween-20 for 1 h, membranes were incubated with primary antibodies overnight followed by TBST washes and 2 h HRP-conjugated secondary antibody incubation, with visualization using BeyoECL Star Kit.

For immunostaining, fixed organs (4 % PFA, 20 min) were blocked with 10 % donkey serum/1% Triton X-100 in PBS for 1 h at 37 °C, and incubated with primary antibodies overnight at 4 °C followed by Alexa Fluor-conjugated secondary antibodies. Cell proliferation, nuclei, and stereocilia were visualized using EdU, DAPI, and phalloidin labeling respectively. The detailed description of the Antibodies utilized in this study was provided in Supplementary Table 4.

To investigate protein–protein interactions, we performed Co-Immunoprecipitation (Co-IP). Utricular tissues were lysed in a buffer supplemented with protease and phosphatase inhibitors, and the soluble protein fraction was isolated by high-speed centrifugation. After quantifying protein concentrations with the BCA assay, equal amounts of lysate were incubated overnight with either a primary antibody against BMP4 or an isotype control. Protein A/G magnetic beads were then used to pull down the resulting immune complexes. The beads were washed extensively to minimize non-specific binding before the captured proteins were eluted and analyzed by Western blot. For the analysis, GAPDH served as a loading control, and membranes were probed with an anti-phospho-Smad1/5/8 antibody to identify specific interactors.

Confocal imaging was performed on Leica SP8/STELLARIS STED microscope, sampling multiple fields across striolar and extrastriolar regions. Hair cells (HCs) and supporting cells/epithelial non-hair cells (SCs/ENHCs) were quantified using ImageJ. Statistical analyses used GraphPad Prism 6.0. The normality of data distributions was evaluated using the *Shapiro–Wilk* test, and homogeneity of variances was assessed with *Levene’s* test. When both assumptions were satisfied, one-way ANOVA was performed, followed by *Tukey’s post*-*hoc* test for multiple comparisons. For datasets that violated either assumption of normality or homogeneity, the non-parametric *Kruskal*–*Wallis* test was applied, with Dunn’s post-hoc test used to assess pairwise differences. Data are presented as mean ± S.E.M., with significance at *p* < 0.05 and high significance at *p* < 0.01. The abbreviations used in the article were summarized in Supplementary Table 5.

## Results

### ENHCS in both developing and damaged utricles are proliferative and can be reprogrammed into HCs with exogenous BMP4

The cellular landscape of the utricular sensory epithelium comprises hair cells (HCs) and a diverse population of non-sensory cells (ENHCs), which encompasses supporting cells (SCs) and transitional epithelial cells [4,47]. We first performed a single-cell RNA sequencing (scRNA-seq) analysis of mouse utricular sensory epithelium at E14 and P2 together with importing publicly available scRNA-seq data of the mouse inner ear at E14 from the gEAR database [48] in order to determine BMP4 expression levels during development. High levels of *Bmp4* expression were seen in the sensory region during early inner ear formation at the embryonic stage, while BMP4 expression gradually decreased in the sensory epithelium after birth. Temporal trajectory analysis suggested that BMP4 is involved in early HC precursor development [[28], [29], [30], [31],^49^]. BMP4 was found in a subtype of ENHC cytoplasm and was upregulated following gentamicin damage (Fig. S2). This raised the question as to whether BMP4 signaling is necessary for ENHC reprogramming into new HCs.

**Fig. 1:**
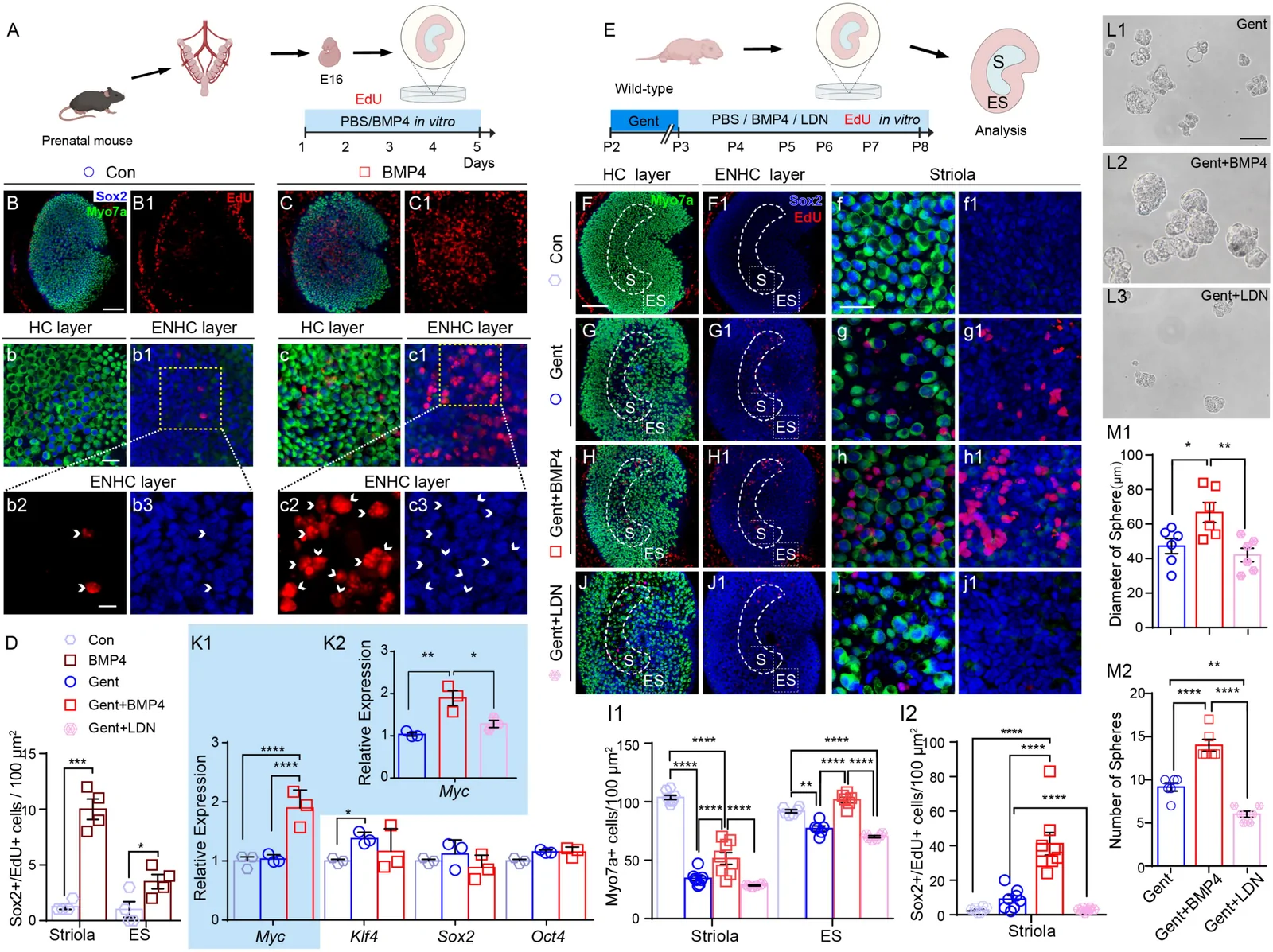
BMP4 is crucial for inner ear progenitor cell proliferation and HC regeneration. (A) Schematic procedure of embryonic mice. Mice were sacrificed on Day 16 of pregnancy, and utricles of embryos were isolated for culture with PBS or BMP4. (B-D) A significant increase in the number of Sox2^+^/EdU^+^ cells (white arrowheads) was observed in the BMP4 group, as measured by cell counts. (E) Schematic procedure in P2 mice. Utricles were isolated from wild-type mice and then cultured with small molecules following gentamicin damage. (F-f1) The HCs were evenly distributed in the striola and extrastriola and had relatively low proliferation rates under non-injury conditions. (G-g1) Post-damage, HC loss and an increase in the number of Sox2^+^/EdU^+^ cells was observed in the striola. (H-h1) An increase in Sox2^+^/EdU^+^ cells in the striola and Myo7a^+^ cells in both regions was observed after the addition of BMP4. (I1-I2) Quantification of the cell numbers from panels F, G, H, and J. (J-j1) The numbers of Sox2^+^/EdU^+^ cells in the striola and Myo7a^+^ cells in both regions were decreased with LDN-193189. (K1-K2) *Myc*, *Klf4*, *Sox2*, and *Oct4* mRNA levels were assayed by real-time qPCR. (L1-L3) Representative images of spheres. (M1-M2) Quantification of the sphere numbers and sphere diameters. Data are presented as the mean ± S.E.M. Unpaired *t*-test in D, two-way ANOVA in J1 and M1, and ordinary one-way ANOVA in J2, L1-L2, and M2. * *p* < 0.05, ** *p* < 0.01, *** *p* < 0.001, **** *p* < 0.0001. Scale bars: 100 μm in B and F; 50 μm in L1; 20 μm in b and f; 10 μm in b1.

We next added exogenous BMP4 to E16 utricles, and we found that BMP4 significantly enhanced the proliferation of ENHCs (Fig. 1A–D, white arrowheads). Similarly, in the gentamicin-damaged utricles (Fig. 1E) exogenous BMP4 led to increased numbers of Sox2^+^/EdU^+^ cells in the striola and the greater numbers of Myo7a^+^ cells in the whole utricle (Fig. 1, Fig. 1; I1-I2). Conversely, blocking of BMP4 expression using LDN-193189 [24,50] decreased the numbers of proliferated ENHCs and Myo7a^+^ HCs (Fig. 1, Fig. 1). The expression level of the transcription factor *Myc*, one of the OSMK factors (*Oct4*, *Sox2*, *Myc* and *Klf4*, the core markers of stem cells) [51,52] was significantly increased with exogenous BMP4 (Fig. 1K1) [53] and decreased by LDN-193189 (Fig. 1K2). Moreover, BMP4 promoted the neurosphere number and diameter (Fig. 1L1-M2), indicating that cell proliferation was enhanced. These results indicated that BMP4 is critical for ENHC proliferation and reprogramming.

### The synergistic reprogramming effect of BMP4 with Notch or WNT signaling

In the mammalian cochlea, the co-activation of *Myc* and *Notch1* reprograms SCs and facilitates HC regeneration [54]. Because *Myc* was increased following BMP4 treatment in the damaged utricle, we continued our study by investigating the synergistic effect of BMP4 with Wnt or Notch signaling on reprogramming.

**Fig. 2:**
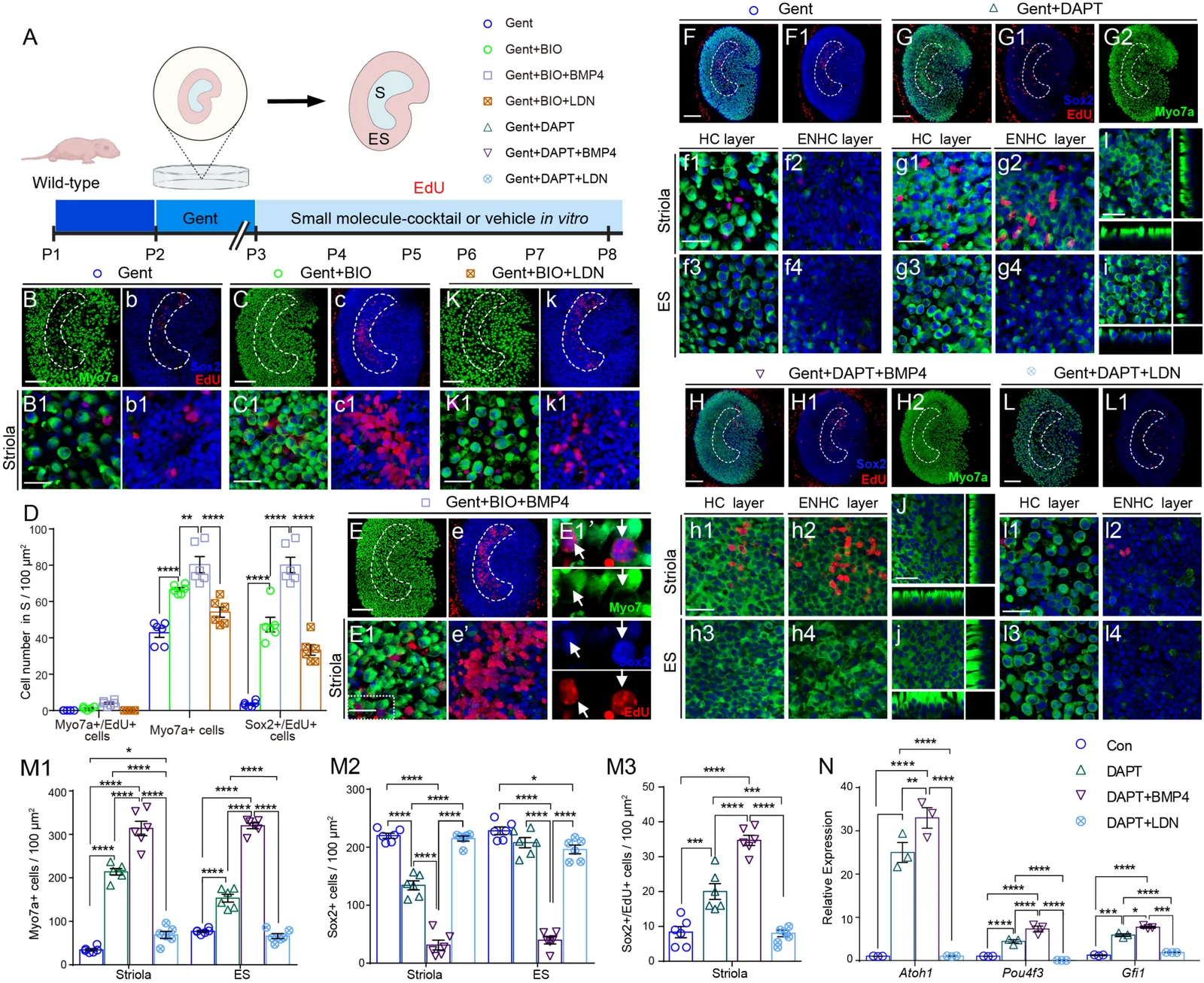
BMP4 enhances utricular progenitor cell reprogramming. (A) Schematic of the experimental procedure. Utricles were isolated from wild-type mice, subjected to gentamicin treatment, and subsequently cultured with the small molecule cocktail. (B-b) Low-magnification images of utricles treated with gentamicin alone (control) in (B) the HC layer and (b) the ENHC layer. Few EdU^+^ cells were detected in the striola. (B1-b1) High-magnification images of the utricular striolar region in the control group in (B1) the HC layer and (b1) the ENHC layer. EdU labeling colocalized with Sox2 in the nuclei. (C-c) Low-magnification images of utricles treated with gentamicin and BIO in (C) the HC layer and (c) the ENHC layer. A notable increase in EdU^+^ cells was observed. (C1-c1) High-magnification images of the utricular striola treated with gentamicin and BIO in (C1) the HC layer and (c1) the ENHC layer. (D) Quantification of cell numbers in the striola. (E-e) Low-magnification images of utricles treated with gentamicin, BIO, and BMP4 in (E) the HC layer and (e) the ENHC layer. There was a significant increase in Sox2^+^/EdU^+^, Myo7a^+^, and Myo7a^+^/EdU^+^ cells in the striola compared to the BIO group. (E1-e1) High-magnification images of the utricular striola in the Gent + BIO + BMP4 group in (E1) the HC layer and (e1) the ENHC layer. (E') A notable number of Myo7a^+^/EdU^+^ cells were observed in the striola (white arrows). (F-f4) In the gentamicin control group, few EdU^+^ cells were detected in the striola. (G-g4) In the Gent + DAPT group, a significant number of Myo7a^+^ cells were observed in the striola, along with an increase in Sox2^+^/EdU^+^ and Myo7a^+^/EdU^+^ cells. (H-h4) When BMP4 was combined with DAPT, a large number of Myo7a^+^ cells were observed across both regions, with only a few remaining Sox2^+^/Myo7a^-^ cells in the entire epithelial area. (I-i) In the Gent + DAPT group, the boundary between the HC layer and ENHC layer was clearly visible in the extrastriola. (J-j) In the Gent + DAPT + BMP4 group, two or even three layers of HCs were observed in the sensory epithelium, and the boundary between ENHCs and HCs was no longer distinguishable. (K-k) Low-magnification images of utricles treated with LDN-193189 and CHIR-99021 following gentamicin damage in (K) the HC layer and (k) the ENHC layer. There was a reduction in the number of Sox2^+^/EdU^+^ and Myo7a^+^ cells. (K1-k1) High-magnification images of utricles treated with LDN-193189 and CHIR-99021 after gentamicin damage in (K1) the HC layer and (k1) the ENHC layer. (L-l4) Treatment with LDN-193189 led to a significant decrease in Myo7a^+^ and Sox2^+^/EdU^+^ cells in both the striola and extrastriola, while the number of Sox2^+^ cells increased. (M1-M3) Quantification of cell numbers across different conditions. (N) qPCR analysis of *Atoh1*, *Gfi1*, and *Pou4f3* expression. Data are presented as the mean ± S.E.M. Statistical analysis: Two-way ANOVA in D, M1-M2, and N; one-way ANOVA in M3. *p < 0.05, **p < 0.01, ***p < 0.001, ****p < 0.0001. Scale bars: 100 μm in B, C, E, and K; 50 μm in F, H, and L; 20 μm in B1, C1, E1, f1, g1, h1, I, J, K1, and l1.

P2 wild-type utricles were isolated and cultured with gentamicin and then treated with BMP4, the Wnt agonist (6-bromoindirubin-3′-oxime (BIO)), or the Notch inhibitor N-{N- [2-(3,5-Difluoro-phenyl)acetyl]-(S)-alanyl}(−(S)-phenyl-glycine *tert*-butyl ester (DAPT, a potent and selective γ-secretase inhibitor [15,55]) along with EdU to label proliferating cells (Fig. 2A). As previously reported, Wnt activation protects against neomycin-induced HC damage [56], and the number of HCs increased after the addition of BIO compared to the control group (Fig. 2B1-c1; D). The number of Sox2^+^/EdU^+^ cells and Myo7a^+^ cells significantly increased in the striola in the BIO + BMP4 group compared to the BIO group (Fig. 2, Fig. 1; E-e1; F). Specifically, the striola in the BIO + BMP4 group showed Myo7a^+^/EdU^+^ cells (Fig. 2, Fig. 1′; white arrows). The addition of DAPT increased the number of Myo7a^+^ cells as well as the number of Sox2^+^/EdU^+^ and Myo7a^+^/EdU^+^ cells in the striola (Figs. 2F-g4). The number of Myo7a^+^ cells increased significantly in the BMP4 + DAPT group in both the striolar and extrastriolar regions, with only a few Sox2^+^ cells remaining (Fig. 2, Fig. 4). A 3D reconstruction further revealed that DAPT + BMP4 eliminated the boundary between HCs and ENHCs, resulting in the presence of two or three HC layers (Fig. 2I-i; J-j).

Silencing of BMP4 using LDN-193189 in conjunction with either BIO or DAPT led to a reduction in the number of Sox2^+^/EdU^+^ cells and Myo7a^+^ cells in the striola (Fig. 2D; K1-k1), while LDN + DAPT also reduced the number of Myo7a^+^ cells in the extrastriola (Figs. 2L-l4, M1-M3). Additionally, the expression levels of early HC markers *Atoh1*, *Gfi1*, and *Pou4f3* [57], [58], [59] were dramatically increased in the DAPT and BMP4 + DAPT groups but decreased following LDN-193189 treatment (Fig. 2N).

Next, we generated *Sox9*-CreER^T^ [2]; ROSA26^tdTomato^; Notch1^flox/flox^ mice to delete Notch1 in neonatal Sox9^+^ cells (Fig. S3A). LDN-193189 significantly decreased the numbers of Myo7a^+^/*Sox9*TM^+^ and *Sox9*TM^+^/EdU^+^ cells in the striola when *Notch1* was knocked out (Fig. S3B-D). These results collectively demonstrate that BMP4 is required for the reprogramming of utricular ENHCs in response to both Wnt and Notch signal stimulation.

### c-Fos is downstream of BMP4 activation-mediated reprogramming

**Fig. 3:**
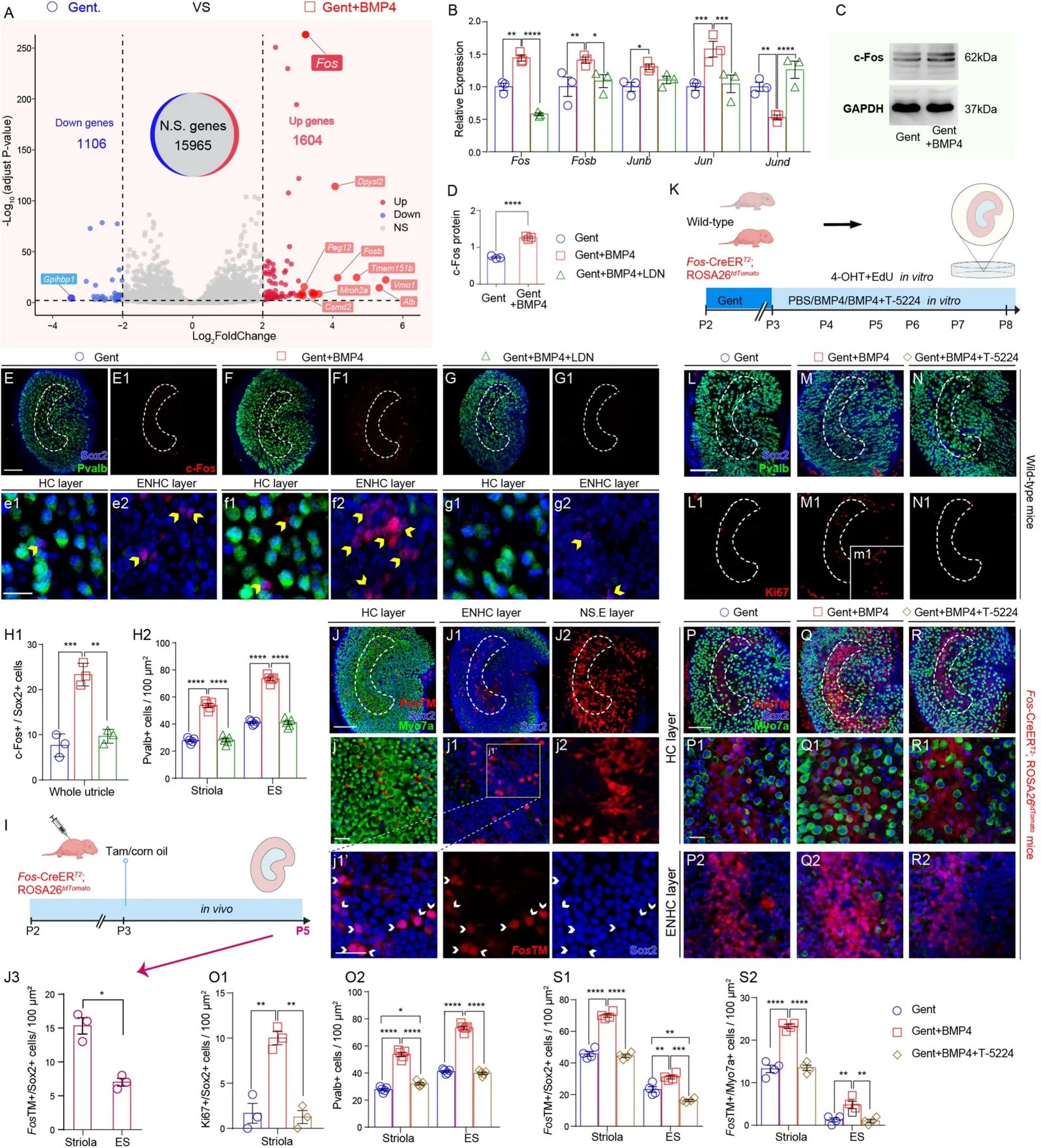
BMP4 promotes inner ear progenitor cell reprogramming by activating the AP-1/c-Fos transcriptional axis. (A) A comparative transcriptomic analysis between the Gent-treated and Gent + BMP4-treated groups. *Fos* and other genes encoding AP-1 protein subunits were significantly up-regulated in the Gent + BMP4 group. (B) Relative mRNA expression levels of AP-1-related transcription factors following BMP4 regulation. (C-D) Western blots and quantification of c-Fos protein levels in the Gent and Gent + BMP4 groups. (E-e2) Immunostaining showing c-Fos expression in the nuclei of ENHCs (yellow arrowheads) following gentamicin-induced damage. (F-g2) Increased c-Fos expression in the Gent + BMP4 group compared to a decrease in the Gent + BMP4 + LDN-193189 group. (H1-H2) Quantification of c-Fos^+^ ENHCs and HCs from panels C–G. (I) Schematic diagram of the experimental design using *Fos*CreER*^T2^*; *ROSA26^tdTomato^* mice. Neonatal mice were injected with tamoxifen or vehicle on P3 and sacrificed on P5 for further analysis. (J-j1′) Post-tamoxifen injection, *Fos*^tdTomato^ (TM) mostly overlapped with Sox2 in the striolar ENHC layer (white arrowheads) and (J2-j2) in the non-sensory epithelial area. (J3) Quantification and comparison of the number of *Fos*TM^+^/Sox2^+^ cells. (K) Schematic diagram of the BMP4 and BMP4 + T-5224 experimental procedure *in vitro*. (L-N1) The numbers of Ki67^+^/Sox2^+^ cells increased with BMP4 and decreased with BMP4 + T-5224. (m1) The magnified version of M1, showing Ki67^+^ cells. (O1-O2) The counts of Pvalb^+^ cells and Ki67^+^/Sox2^+^ cells across the three groups. (P-P2) After 5 days of tracing, *Fos*TM was mainly expressed in the ENHC layer of the striola. (Q-R2) *Fos*TM expression was increased in the BMP4 group and decreased in the BMP4 + T-5224 group. (S1-S2) Quantification and comparison of the cell numbers. Data are presented as the mean ± S.E.M. Unpaired *t*-test in D and J3. Two-way ANOVA in H2, O2, and S1-S2 and ordinary one-way ANOVA in H1 and O1. *p < 0.05, ** *p* < 0.01, *** *p* < 0.001, **** *p* < 0.0001. Scale bars: 100 μm in E, L, J, and P; 20 μm in c1, j1, j1′, and P1.

To comprehensively study the molecular nature of BMP4 functions in ENHC reprogramming into HCs, we profiled the bulk RNA-seq to assess changes in downstream transcriptional networks. The sequencing data revealed that 2710 genes were differentially expressed in the BMP4-treated group, with 1604 genes being upregulated and 1106 being downregulated. Notably, *Fos*, an immediate early gene [60] that rapidly and transiently responds to various extracellular stimuli [61], showed a significant increase following BMP4 treatment (Fig. 3A–D). Exogenous BMP4 triggered the nuclear accumulation of c-Fos in ENHCs (Fig. 3, Fig. 2; F-f2; yellow arrowheads), while LDN-193189 resulted in a decreased number of c-Fos^+^/Sox2^+^ cells (Fig. 3, Fig. 2). Furthermore, the *Fos*-CreER*^T2^*; ROSA26*^tdTomato^* transgenic mouse line also verified the expression of c-Fos in the vestibular sensory epithelium (Fig. 3I; 3J-J3; white arrowheads). The temporal changes in c-Fos expression suggest that c-Fos is activated during HC regeneration.

c-Fos, a key member of the AP-1 family, is essential for both stem cell reprogramming and neural cell regeneration [62], [63], [64]. To determine whether c‑Fos mediates the effects of BMP4 on ENHC reprogramming and its sensitization to Notch/Wnt signaling, we performed a rescue experiment using T‑5224 (10 μM), a selective AP‑1/c‑Fos inhibitor) [65]. When c‑Fos activity was inhibited by T‑5224, exogenous BMP4 failed to promote either ENHC proliferation (Ki67^+^/Sox2^+^) or reprogramming (Parvalbumin^+^) compared with the BMP4‑only group (Fig. 3K; L-N1; O1-O2). Similarly, treatment with T‑5224 significantly reduced the number of Sox2^+^/EdU^+^ cells in the striolar region relative to the BMP4 + BIO condition. Co‑culture with T‑5224 also decreased both Myo7a^+^ cells and Sox2^+^/EdU^+^ cells in the utricle compared with the BMP4 + DAPT group (Fig. S4). In addition, BMP4 treatment markedly increased *Fos*TM expression, an effect that was reversed by T‑5224 (Fig. 3 K; P-R2; S1-S2). These results indicate that inhibition of c‑Fos activity attenuates the pro‑reprogramming effects of BMP4 on ENHCs. Thus, AP‑1/c‑Fos acts as a critical downstream mediator of BMP4 signaling, facilitating ENHC reprogramming and enhancing their responsiveness to Notch and Wnt pathways.

**Fig. 4:**
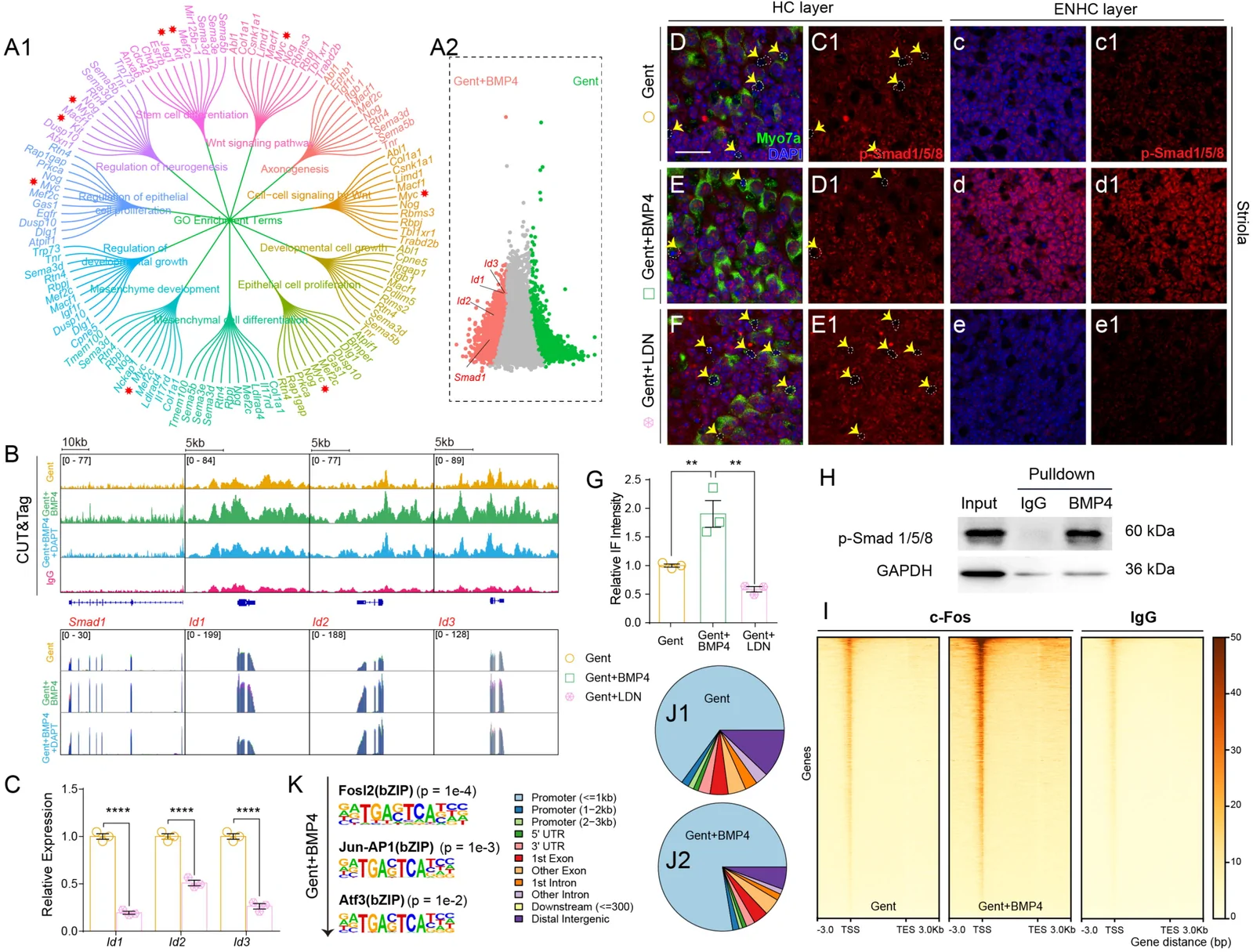
BMP4/c-Fos axis induced *Smad*/*Id* signaling activation. (A1) Gene ontology enrichment and cnetplot analysis for visualizing the predicted targets of up-regulated c-Fos binding peaks in the Gent + BMP4 group compared to the Gent group. (A2) MA plots for differential c-Fos binding peaks between the Gent group and the Gent + BMP4 group. (B) The Integrative Genomics Viewer screenshot showing expression peaks and c-Fos CUT&Tag binding peaks for representative regions around *Smad1*, *Id1*, *Id2*, and *Id3* in the utricle. (C) Relative mRNA expression of *Id1-3* following treatment with LDN-193189. (D-c1) In the Gent group, phosphorylated Smad1/5/8 (p-Smad1/5/8) was detected in the cell nuclei. (E-f1) The immunofluorescence intensity of p-Smad1/5/8(yellow arrows) increased in the Gent + BMP4 group (E-e1) and decreased in the Gent + LDN group (F-f1). (G) Semi-quantification of p-Smad1/5/8 immunofluorescence across groups. (H) Co-IP validation of BMP4 interacting proteins from the p-smad1/5/8. (I) Heatmap of CUT&Tag peaks in a 3 kb region upstream from the transcription start site (TSS) and 3 kb downstream from the transcription end site (TES) in the groups. (J1-J2) Pie chart of the c-Fos CUT&Tag binding region genomic annotations in the Gent and Gent + BMP4 groups. (K) Enriched motifs identified by HOMER in the Gent + BMP4 group. Data are presented as the mean ± S.E.M. Ordinary one-way ANOVA in F, and two-way ANOVA in G. ** *p* < 0.01, **** *p* < 0.0001. Scale bars: 20 μm in B.

To further elucidate the transcriptional mechanisms underlying BMP4‑mediated reprogramming, we performed Cleavage Under Targets and Tagmentation (CUT&Tag) sequencing using an anti‑c‑Fos antibody. A nonspecific IgG sample served as a negative control to validate the specificity of the assay. Differential peak analysis between groups, conducted using edgeR [66], revealed that BMP4 treatment led to an increase in 1,206c‑Fos–bound regions and a decrease in 1,649 regions compared to the Gent group. Upregulated peaks were annotated to key genes such as *Myc*, *Kit*, and *Jag1*. Functionally, these peaks were associated with biological processes including cellular reprogramming, proliferation, developmental regulation, neurogenesis, and Wnt signaling. (Fig. 4A1-A2).

Integration of CUT&Tag and RNA‑seq datasets showed elevated expression of the BMP signal transducer *Smad1* [67], [68] and increased transcription of *Id1–3* following c‑Fos binding activation under BMP4 stimulation (Fig. 4A2; B, Fig. S5). Notably, this induction of *Id1–3* was suppressed by LDN‑193189, a selective BMP receptor inhibitor (Fig. 4C). Consistent with these molecular findings, confocal imaging demonstrated that p‑Smad1/5/8 was predominantly cytoplasmic after injury (Fig. 4, Fig. 1, yellow arrowheads), but translocated to the nucleus following BMP4 treatment (Fig. 4, Fig. 1), This translocation was reversed by LDN‑193189 (Fig. 4, Fig. 1; G). Furthermore, co‑immunoprecipitation using an anti‑p‑Smad1/5/8 antibody confirmed a specific interaction between phosphorylated Smad1/5/8 and BMP4 in the mouse utricle (Fig. 4H). Genomic annotation of c‑Fos CUT&Tag peaks indicated that promoter‑region binding was enriched in the Gent + BMP4 group relative to Gent alone (I, J1-J2). Finally, HOMER motif analysis identified significant enrichment of AP‑1 regulatory motifs, suggesting a self‑activation mechanism of c‑Fos during the BMP4‑induced reprogramming process (Fig. 4K).

Interestingly, when combined with DAPT the BMP4-induced c-Fos binding peaks were enriched in terms related to sensory organ development compared to BMP4 alone. Specifically, DAPT enhanced c-Fos binding to *Myo6*, *Gfi1*, and *Atoh1* (Fig. S6) [59].

### HC regeneration by BMP4 analogue cocktail-driven reprogramming in adult mice *in vitro*

Previous studies have reported that chalcone compounds can activate the BMP/Smad1/5 signaling pathway [69]. To simulate the effects of BMP4 protein and to facilitate its clinical translational application, we added either isoliquiritigenin (ISO) or 4′-hydroxychalcone (Hyd) to *ex vivo* cultures of damaged utricles. Compared to the control group, both ISO and Hyd increased the numbers of Sox2^+^/EdU^+^ and Myo7a^+^ cells in the striola and extrastriola. Notably, Hyd exhibited stronger effects than ISO and enhanced ENHC reprogramming in response to the Notch signaling pathway, as evidenced by the increased number of Myo7a^+^ cells in the extrastriola (Fig. S7). Additionally, we compared three activators of the Wnt signaling pathway and found that the CHIR-99021 (CHIR) group showed the most significant increase in the number of Myo7a^+^ cells and Sox2^+^/EdU^+^ cells, surpassing the effects observed in the Wnt3a and BIO groups (Fig. S7).

**Fig. 5:**
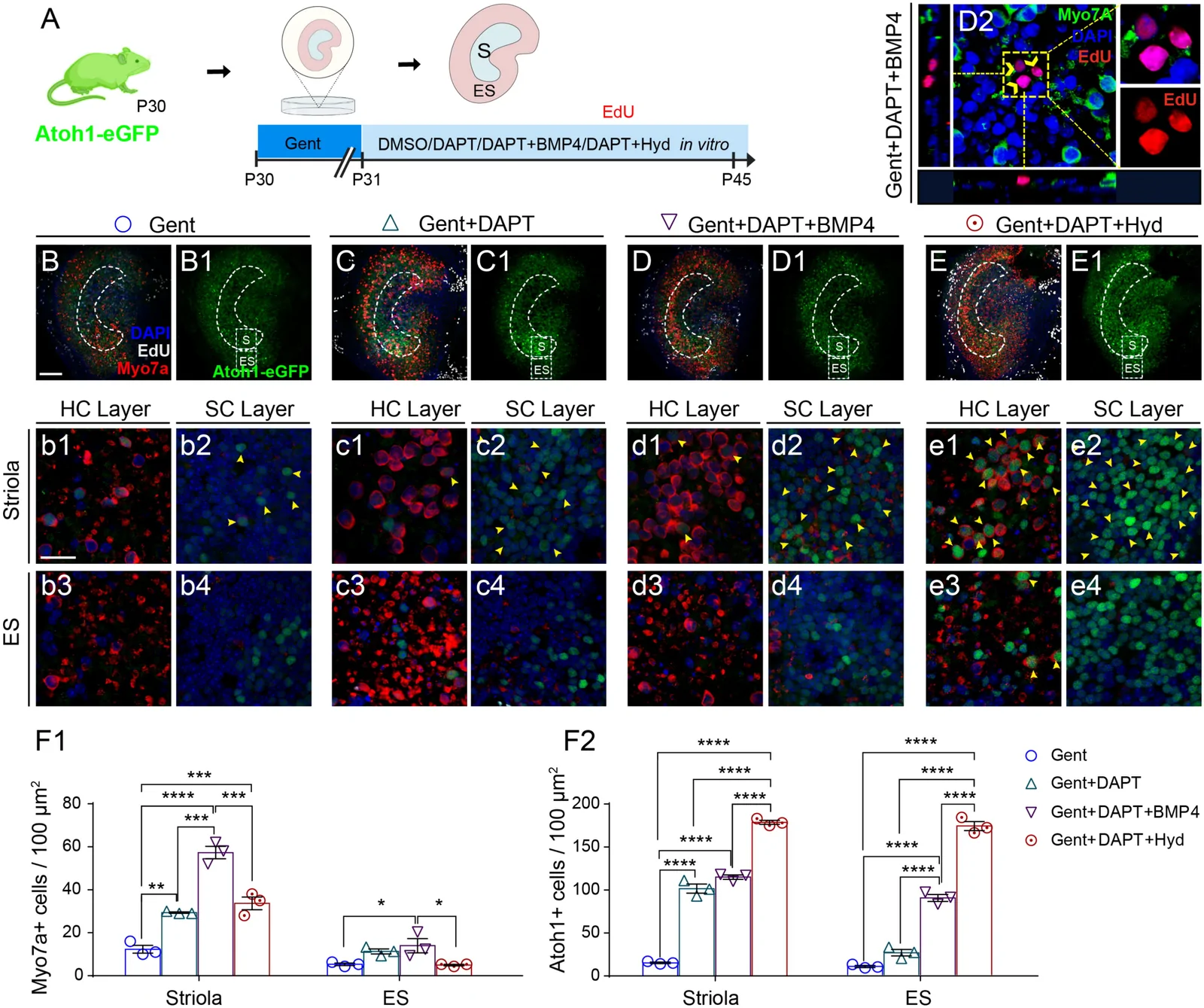
HC reprogramming in adult mice *in vitro*. (A) Schematic diagram of the experimental procedure with adult Atoh1-eGFP transgenic mice *in vitro*. (B-b4) In the Gent group, only a minimal number of Myo7a^+^ cells persisted in the sensory epithelium, with few eGFP^+^ cells (yellow arrows) being observed. (C-c4) In the DAPT group, the quantity of Myo7a^+^ cells doubled compared to the control group. Additionally, the count of eGFP^+^ cells in the SC layer experienced a significant increase in the striola. (D-d4) In the DAPT + BMP4 group, the numbers of both eGFP^+^ cells and Myo7a^+^ cells significantly increased. (D2) EdU^+^ cells (yellow arrowheads) were detected in HC layer in DAPT + BMP4 group. High-magnification images of the EdU^+^ cells. (E-e4) In the DAPT + Hyd group, although Myo7a^+^ cells were slightly increased, a large number of cells in the SC layer co-localized with Atoh1-eGFP in both the striola and extrastriola compared to the Gent + DAPT group. (F1-F2) Quantification and comparison of the cell numbers. Data are presented as the mean ± S.E.M. Two-way ANOVA. *p < 0.05, **p < 0.01, ***p < 0.001, ****p < 0.0001. Scale bars: 100 μm in B; 20 μm in b1.

*Atoh1*, which is maximally expressed during the embryonic stages of development, undergoes rapid downregulation within the first week after birth [70], [71]. Here we used Atoh1-eGFP transgenic mice, which express eGFP-labeled proteins in immature sensory HCs, to investigate ENHC reprogramming and HC regeneration in adult mice. P30 Atoh1-eGFP utricles were cultured with gentamicin and subsequently treated with DAPT (+BMP4/Hyd) for 15 days (Fig. 5A). Treatment with DAPT resulted in an approximately twofold increase in Myo7a^+^ cells within the striola compared with the Gent group, reaching 29.33 ± 0.33 HCs/100 µm^2^ in the striola and 11.00 ± 1.16 HCs/100 µm^2^ in the extrastriola. Concurrently, a substantial increase in eGFP^+^ cells was observed in the SC layer of the striola (Fig. 5, Fig. 4, C-c4, yellow arrowheads). The combination of DAPT and BMP4 further increased the numbers of Sox2^+^/eGFP^+^ and Myo7a^+^ cells (Fig. 5, Fig. 4). Occasional EdU^+^ cells were detected within the HC layer of the DAPT + BMP4 group (Fig. 5D2), suggesting limited proliferative contribution. In contrast, the DAPT + Hyd combination caused only a mild increase in Myo7a^+^ cells, but it significantly expanded the pool of Sox2^+^ cells co‑expressing Atoh1‑eGFP in both the striola and extrastriola (Fig. 5, Fig. 4; F1-F2). These results indicated that activation of the BMP4 pathway, particularly through the agonist Hyd, expands the population of Atoh1–expressing supporting cells and promoted their reprogramming toward HC regeneration.

### HC regeneration and functional vestibular recovery by BMP4 analogue cocktail-initiated reprogramming *in vivo*

**Fig. 6:**
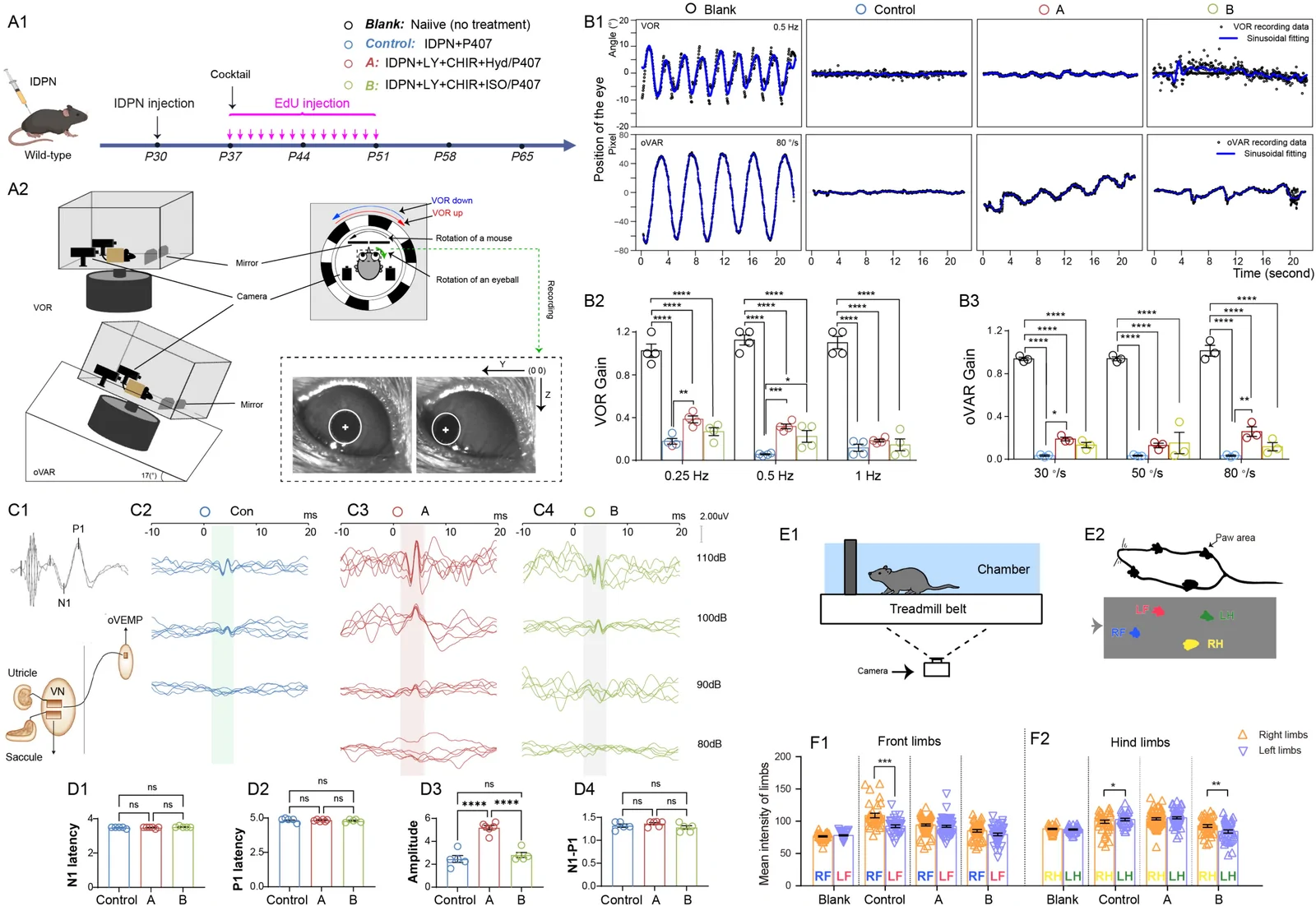
Recovery of vestibular function in adult mice *in vivo*. (A1) Schematic of the experimental procedure. Wild-type mice were unilaterally injected with IDPN on P30, followed by daily EdU injections from P37 to P51. A small molecule cocktail was injected via the round window on P37. (A2) Schematic of the VOR apparatus. Mice were positioned on a platform, with two cameras capturing eye movements during rotations. (B1) Representative trajectories (black dots) of horizontal eye positions during the rotations. (B2-B3) Quantification of HAVOR and OVAR gain across different experimental groups. (C1) Architecture of the oVEMP pathway and its waveform. (C2-C4) A comparison of oVEMP waveforms in control, group A, and group B groups – recorded at both threshold and suprathreshold stimulus levels. (D1-D4) Quantitative analysis of oVEMP parameters including amplitude, N1 latency, P1 latency, and interpeak interval at 110 dB SPL. (E1) Schematic of the CatWalk XT system. Mice were placed on a treadmill with a camera located beneath the treadmill belt to record gait patterns. (E2) A screenshot from the CatWalk XT video illustrating paw prints and gait direction for the left forepaw (LF), left hind paw (LH), right forepaw (RF), and right hind paw (RH). (F1-F2) Post-IDPN injection, a significant difference in mean intensity of the front limbs was observed compared to controls. (F1): Blank n = 31, Control n = 31, A n = 63; B n = 40; (F2): Blank n = 31; Control n = 31, A n = 56, B n = 38. Data are presented as the mean ± S.E.M. Two-way ANOVA in B2 and D1-D4; unpaired *t*-test in F1-F2. *p < 0.05, **p < 0.01, ***p < 0.001, ****p < 0.0001.

LY411575 is a potent and highly selective γ-secretase inhibitor that effectively suppresses Notch signaling, thereby modulating cell fate decisions and promoting hair cell differentiation in the inner ear and other sensory epithelia [72], [73], [74]. To assess functional recovery, we used a thermosensitive 30 % P407 gel to inject LY411575 + CHIR + Hyd/ISO into adult mice following vestibular damage induced by 3,3′-iminodiproprionitrile (IDPN) [75], [76]. This approach optimized sustained drug release and minimized drug loss. IDPN, known for its neurotoxic and vestibulotoxic properties [42], was intraperitoneally injected into P30 mice at a dosage of 5 µl/g to induce vestibular dysfunction. After one week, we administered LY411575 + CHIR + Hyd/P407 (Group A), LY411575 + CHIR + ISO/P407 (Group B), or P407 alone (Control) via the round window membrane. Additionally, we systemically injected EdU (5 mg/ml, 50 μg/g body weight) for two consecutive weeks (Fig. 6A1).

To maintain a stable visual field during head movements, the vestibulo-ocular reflex (VOR) generates compensatory eye movements in the direction opposite to the head's motion (Fig. 6A2). Triggered by the vestibular apparatus in response to sensory acceleration, the VOR is an essential *in vivo* tool for objectively and quantitatively evaluating vestibular function [41], [77]. The VOR gain, which is the ratio of the amplitude or angular velocity between the response and the stimulus, serves as a quantitative measure of vestibular efficacy [41]. At P65, we performed the horizontal angular vestibulo-ocular reflexes (HAVOR) test and the off-vertical axis rotation (OVAR) test to assess the function of the horizontal cristae and macular organs, respectively. In wild-type mice with normal vestibular function, the pupil's angle dynamics in response to sinusoidal reverse rotation stimuli exhibited a sinusoidal pattern across various stimulus frequencies (Fig. 6B1, Blank). However, at 4 weeks after IDPN injection VOR testing did not show any distinct eye movement waveforms in the control group at frequencies of 0.25, 0.5, or 1 Hz (Fig. 6B1, blue, control). In contrast, Group A and Group B showed sinusoidal eye movements, suggesting a partial recovery of vestibular function. Furthermore, the VOR gain in Group A was obviously enhanced at the tested frequencies of 0.25 and 0.5 Hz (Fig. 6B1-B2), indicating an improvement in their vestibular response compared to control. Consistent results were observed in OVAR tests (Fig. 6B3). Functional recovery of the vestibular system was first apparent three weeks post‑treatment (Fig. S8). Electrophysiological evaluation using oVEMP (Fig. 6C1) further corroborated these findings. While response amplitudes increased in both Groups A and B (Fig. 6C2-C4), the enhancement reached statistical significance only in Group A (Fig. 6D1-D4).

Next, we used the CatWalk XT 2020 system to evaluate the gait [78] (Fig. 6E1-E2). Mean intensity (the average pressure exerted by each limb on the floor) was mainly used as the evaluation indicator for mouse vestibular dysfunction. Corresponding with the VOR test results, the gait analysis revealed compromised limb function following IDPN administration. Although there was recovery observed in the front limbs of both groups, only Group A showed improvement in hindlimb function (Fig. 6F1-F2, F1 Control, n = 31, *p* < 0.001; F2 Control, n = 31, *p* < 0.05; F2 B, n = 38, *p* < 0.01). Comprehensive evaluation of HC counts, VOR, oVEMP, and gait parameters (Supplementary Table S6) demonstrated that Cocktail A yielded the most robust and consistent functional recovery, establishing it as the optimal therapeutic combination.

**Fig. 7:**
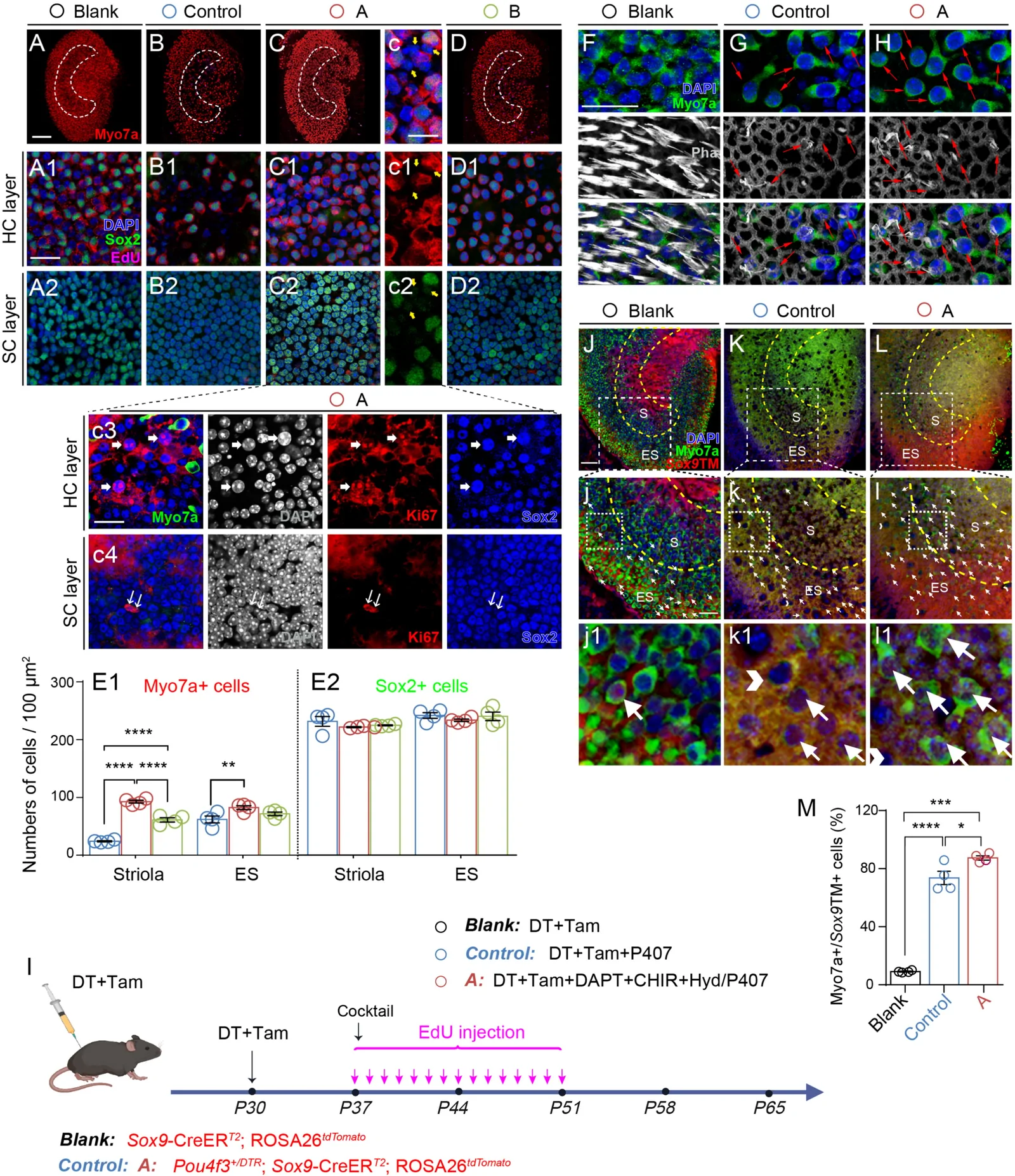
HC regeneration in adult mice *in vivo*. (A1-A2) Images from control mice showing normal HC distribution. (B-B2) Following IDPN treatment, noticeable HC loss was evident in the striola region of the utricles. (C-C2; D-D2) An increase in Myo7a^+^ cells was observed in both Group A and Group B. (c3-c4) Ki67^+^/Sox2^+^ cells (white arrows) were detected in Group A. (E1-E2) Comparison of the cell numbers across groups. (F-H) A notable increase in immature hair bundles was seen in Group A. (I) Experimental procedure schematic of *Pou4f3^+/DTR^*; *Sox9*-CreER*^T2^*; ROSA26*^tdTomato^* mice. Mice received diphtheria toxin (DT) and tamoxifen. (J-j) Sox9-CreER*^T2^*:ROSA26*^tdTomato^* mice that received DT injection but did not possess the *Pou4f3^+/DTR^* gene modification (blank). (K–L) There was a significant increase in Myo7a^+^/*Sox9*TM^+^ cells in Group A compared to the damaged controls (white arrows). (M) Statistical comparison of cell numbers between groups. Data are presented as the mean ± S.E.M. Two-way ANOVA in E1-E2 and one-way ANOVA in M. *p < 0.05, **p < 0.01, ***p < 0.001, ****p < 0.0001. Scale bars: 100 μm in D, M and m; 20 μm in D1 and I.

We conducted further analyses by counting the number of HCs in the utricles from the above groups and found an increased number of Myo7a^+^ cells in both cocktail-treated groups compared to the control group (Fig. 7, Fig. 2; E1-E2). The reprogrammed cells exhibited irregularly shaped and smaller nuclei with weak Sox2 expression, and Group A displayed sparse Sox2^+^ cells that were displaced in an upward direction (Fig. 7, Fig. 2, yellow arrows). To track proliferating cells, systemic EdU administration was performed over a two‑week period. At the study endpoint, no EdU‑labeled SCs were detected, suggesting minimal proliferation. However, a few Ki67^+^/Sox2^+^ SCs were identified in the utricles of adult mice treated with Cocktail A, indicating limited but detectable proliferative activity (Fig. 7c3-c4, white arrows).

To evaluate hair cell (HC) maturation, we stained the actin-rich stereocilia using a fluorescently labeled phalloidin conjugate. The blank group displayed normal hair bundles (Fig. 7F). However, there was an increase in the number of HCs with immature stereocilia bundles in Group A compared to the control group (Fig. 7G-H, red arrows), which indicated that the small molecule cocktail-initiated SC reprogramming into HCs.

Furthermore, we fate-mapped SCs in the *Pou4f3^+/DTR^*; *Sox9*CreER*^T2^*; ROSA26*^tdTomato^* mice to investigate the properties of reprogrammed HCs in the damaged utricle. After ablation of HCs with diphtheria toxin at P30 and subsequent activation of Cre recombinase with tamoxifen (Fig. 7I), we observed a significant increase in fate-mapped Myo7a^+^ cells in the striolar regions of Group A compared to the controls (Fig. 7J-M). These findings suggest that Sox9^+^ SCs contribute to reprogrammed HCs through the coordinated regulation of the BMP4, Wnt, and Notch signaling pathways.

## Discussion

The loss of vestibular HCs can arise from various causes, including exposure to ototoxic chemicals, viral infections, aging, or genetic factors, and vestibular HCs possess a limited capacity for regeneration following damage. Direct reprogramming techniques have demonstrated significant success in converting SCs into HCs during the neonatal phases, although these strategies have shown limited efficacy in adults [54], [79]. Notably, a member of transforming growth factor family (TGF-α) has proven effective in restoring HCs in adult mouse vestibular organs post-injury *in vitro*. [6].

BMP4, another member of the TGF-β superfamily, is crucial for the differentiation, proliferation, and maturation of stem cells and plays essential roles in the development and healing of diverse tissues [80], [81]. It has been repeatedly reported that BMP4 induces cell reprogramming to regenerate other types of cells [24], [82], [83], [84], but the role and molecular mechanism of BMP4 in inner ear reprogramming and HC regeneration remain unclear. In the context of the inner ear, BMP4 plays a pivotal role in guiding sensory progenitor cells to differentiate into HCs during embryonic development [21], [22], [27], [30], [85]. In post‑hatch chick models, BMP4 was identified as one of the genes showing the most pronounced expression differences between cochlear and utricular epithelia, suggesting region‑specific regulatory functions [86]. Notably, comparative studies indicate that the functional roles of BMP4 may vary across species, as findings in mice and chicken embryos show divergent signaling outcomes [30], [85], [87], while its function in primates and other higher mammals has yet to be elucidated. Here we explored the regenerative potential of BMP4 and its analogs by exploring c‑Fos‑mediated reprogramming pathways. The administration of the BMP4-based cocktail following vestibular damage not only improved HC numbers, but also enhanced vestibular function in adult mice *in vivo* as evidenced by the VOR and gait analysis, which aligns with previous findings that correlate HC regeneration with functional recovery in the vestibular system [88].

Several transcription factors are involved in the process of exogenous BMP4 and promote reprogramming in the vestibular system, including *Myc* family genes, the HC competence transcription factor *Atoh1* either alone or in combination with other HC-specific transcription factors such as *Pou4f3* or *Gfi1*, and *Fos* family genes. c-Myc has been reported to be involved in SC reprogramming in the cochlea [54]. Previous research has demonstrated that transient expression of c-Myc can create a fate-restricted cell line that is otic-fate restricted, self-renewing, and capable of differentiating into functional HCs and neurons [89]. In the present study, we observed that *Myc* emerged as a key gene among stem cell markers and reprogramming process, showing increased expression following BMP4 treatment in the damaged utricle (Fig. 1M1-M2), with these target genes being influenced by c-Fos activation as confirmed by our CUT&Tag analysis (Fig. 4A1).

The AP-1 complex is classically recognized as a family of transcription factors associated with cell proliferation, differentiation, and oncogenesis. However, recent evidence reveals that AP‑1 members exert diverse, and occasionally opposing, roles during cellular reprogramming [90], [91]. For instance, c‑Jun, a core AP‑1 component, functions as a barrier to somatic cell reprogramming, as its depletion markedly enhances the efficiency of OSKM‑mediated induced pluripotent stem cell (iPSC) generation [92]. In contrast, c‑Fos, another AP‑1 subunit, can in specific contexts facilitate reprogramming or modulate stem cell senescence and tumor cell proliferation [93], [94]. Furthermore, its paralog Fosl1 has recently been identified as a novel reprogramming factor in tumorigenesis, underscoring the versatility of AP‑1 family members in regulating cell fate transitions [95], [96].

In the current study, we reveal a previously unrecognized role for **c-Fos**—a transcription factor and component of the AP-1 complex—in cellular reprogramming within the utricular epithelium [97], is well-known for initiating critical cellular processes, including cell cycle entry, proliferation, and regeneration [63]. As an early response gene and transcription factor in the nervous system, c-Fos is a protein whose importance has often been overlooked. Beyond its role in the AP-1 pathway, c-Fos also performs additional functions, and it associates with endoplasmic reticulum membranes and stimulates lipid synthesis by interacting with specific lipid-synthesizing enzymes [98], which is also supportive of neuronal differentiation in the nervous system [99]. Some studies have detected c-Fos expression in the cochlea following acoustic trauma, suggesting that it plays a role in the survival or protection of cells in the organ of Corti [100]. Notably, previous developmental studies have proposed AP‑1 as a downstream effector of BMP4 signaling in Xenopus embryos [101]. Consistent with this, we identified Fos as one of the most significantly upregulated genes following BMP4 treatment in our model. Lineage‑tracing experiments demonstrated that *Fos*TM^+^ HCs emerged after BMP4 exposure (Fig. 3P-S2), suggesting that these newly differentiated HCs likely originated from Fos^+^ progenitors. Further analyses, including bulk-RNA sequencing and c-Fos CUT&Tag to examine peak activations, confirmed that in the presence of BMP4 genes such as *Id*s and *Smad1* are downstream of c-Fos activation. This result is supported by the finding that GO terms related to the proliferative capacity of the epithelium, stem cell differentiation, Wnt signaling, and epithelial cell proliferation were particularly enriched in the BMP4-treated group (Fig. 4). These findings led us to propose that the activation of c-Fos post-injury might be related to the reprogramming of vestibular ENHCs, thus facilitating HC regeneration.

In addition, our findings further demonstrate that BMP4 is required for enhancing the role of Notch inhibition or Wnt activation in reprogramming by initiating c-Fos. In the presence of Notch inhibition, BMP4 influences the genes that are pivotal for HC development, including *Atoh1* [57], *Gfi1*, and *Pou4f3*, the transcription factors that are important for HC fate determination [82]. LDN-193189, a dorsomorphin derivative that broadly suppresses BMP transcriptional activity [102], diminished the regenerative response to DAPT, indicating that BMP4 signaling is a crucial component for the efficacy of Notch inhibition-mediated HC regeneration (Fig. 2). Simultaneously, in conjunction with the enrichment analysis results of c-Fos CUT&Tag peaks, we found that DAPT + BMP4-induced activation of c-Fos is closely related to sensory organ morphogenesis and synapse organization.

The anatomical isolation of the inner ear presents a significant obstacle to effective drug delivery, as therapeutic molecules administered systemically must traverse the blood–labyrinth barrier. This often results in subtherapeutic intra‑cochlear drug levels and increased systemic exposure [103]. In this context, the application of small-molecule cocktails to promote cellular reprogramming has emerged as a promising strategy for HC regeneration and the restoration of vestibular function in adult mammals [54]. Small molecules have several advantages; they can penetrate cells directly, bypassing the need for complex delivery methods such as transfection or viral vectors, and they avoid the genomic integration risks associated with these vectors. Furthermore, small molecules are characterized by their diverse structures, precise targeting, high selectivity, and affordability, making them appealing for both research and clinical use. In this study, we employed a poloxamer P407-based thermosensitive hydrogel system [39], [76], [104] for the sustained local release of a small-molecule cocktail to initiate HC regeneration in mice via the round window membrane. Despite these promising results, inner ear pharmacotherapy remains an early‑stage field, and the clinical translation of small‑molecule–based strategies demands careful evaluation [105]. Foremost among these considerations are potential off‑target effects, as pathway modulators and agonists could inadvertently trigger proliferative responses in non‑sensory tissues [106]. Likewise, the possibility of teratoma formation or uncontrolled hyperplasia constitutes a major safety concern, necessitating comprehensive toxicological and dose‑response assessments [72], [106]. Moreover, the pharmacokinetic and pharmacodynamic profiles of small molecules within the inner ear environment remain poorly characterized and warrant further study [107]. Finally, while our data demonstrate robust HC regeneration and partial functional recovery, the long‑term viability of newly generated HCs and the potential depletion of the supporting cell population require continued investigation.

Our study has several limitations that should be considered and addressed in future investigations. First, due to current technical constraints, we were unable to determine definitively whether BMP4 activation influences additional members of the AP‑1 transcription factor family beyond Fos. Such information would be valuable for clarifying the breadth of transcriptional regulation underlying BMP4‑mediated reprogramming. Second, our evaluation of vestibular functional recovery remains limited. Although we observed the regeneration of HCs, we were not able to perform ultrastructural analyses to assess the morphology and integrity of stereociliary bundles in the striola, nor could we conduct in‑depth electrophysiological or behavioral assessments to determine the functional performance of the regenerated HCs. Third, the intrinsic constraints of our animal model prevented more comprehensive and longitudinal evaluations of vestibular recovery.

Additionally, it is important to acknowledge the potential off‑target and safety concerns associated with small‑molecule modulation. While the small-molecule, either Hyd or ISO effectively promoted HC regeneration, these agents may also activate unintended signaling pathways or induce proliferative responses in non‑sensory tissues, necessitating cautious interpretation and further toxicological assessment. Finally, the long‑term survival, integration, and stability of newly regenerated HCs remain uncertain. Extended follow‑up studies will be required to determine whether these cells maintain normal structure and function over time and whether continuous signaling manipulation might compromise the supporting cell pool or inner ear homeostasis.

Despite these limitations, our findings contribute significantly to the molecular understanding of SC reprogramming and HC regeneration. By uncovering the intricate interplay between BMP4, Wnt/Notch signaling pathways, and c-Fos activation, we have established a critical framework for developing innovative regenerative therapies that can potentially restore hearing and balance.

## Compliance with ethics requirements

All Institutional and National Guidelines for the care and use of animals (fisheries) were followed.

## CRediT authorship contribution statement

**Dan You:** Investigation, Writing – original draft. **Yunzhong Zhang:** Investigation, Formal analysis, Writing – original draft. **Kunkun Wang:** Investigation. **Luo Guo:** Methodology. **Jin Guo:** Investigation. **Chenhao Che:** Investigation. **Wen Li:** Validation. **Liping Zhao:** Validation. **Huawei Li:** Conceptualization, Writing – review & editing, Funding acquisition, Resources, Supervision. **Shan Sun:** Conceptualization, Formal analysis, Writing – review & editing, Funding acquisition, Resources, Supervision.

## Declaration of competing interest

The authors declare that they have no known competing financial interests or personal relationships that could have appeared to influence the work reported in this paper.
