# Supplementary material for: BMP4 cocktail promotes utricular progenitor reprogramming and vestibular functional recovery in adult mice
Source: J Adv Res. 2025 Nov 29;87:523–38. doi: 10.1016/j.jare.2025.11.065 (PMC13539158; doi:10.1016/j.jare.2025.11.065)
Supplement: Supplementary file 1 [file mmc1.docx]

**Supplementary figure legends.**

**Figure S1: Dose-response curves and the determination of optimal concentrations for various reagents.**

Dose-response curves were generated for isoliquiritigenin (ISO) (A), 4'-hydroxychalcone (Hyd) (B), BMP4 (C), T-5224 (D), and LDN-193189.

(E) in treated utricles. EC_50_ curves were used for BMP4, ISO, and Hyd, while IC_50_ curves were used for T-5224 and LDN-193189. The optimal drug concentration was determined based on the counting of Myo7a^+^ cells and Sox2^+^/EdU^+^ cells, which served as indicators of drug effect. The shaded purple region represents the identified optimal concentration range for each drug, and the corresponding calculated values are presented in the respective panels for the specific chemical. *Abbr:* LDN: LDN-193189; ES: extrastriolar; Hyd: 4'-hydroxychalcone; ISO: isoliquiritigenin; Pvalb: parvalbumin; con. concentration; EC: effective concentration; IC: inhibitory concentration.

**Figure S2: *Bmp4*^+^ progenitors are found in both developing and damaged utricular epithelia.**

(A1) Immunofluorescent staining for Myo7a and Sox2 in the utricle.

(A2) Diagram of the sensory epithelial cell layer (including the HC layer and ENHC layer).

(B1) Single-cell RNA sequencing (scRNA-seq) of the inner ear (from gEAR database) at E14. Distinct cell clusters are denoted by varying colors.

(B2) Expression levels of *Sox2*, *Atoh1*, and *Bmp4* at E14. Notably, *Bmp4* exhibited high expression in utricular sensory epithelium cells (indicated by red arrow).

(C1) scRNA-seq of sensory epithelial cells at E14 in the mouse utricle, with cell clusters indicated by different colors.

(C2) The Slingshot trajectory illustrating the ordering of single cells in pseudotime.

(C3) Expression levels of E14 mouse utricular sensory epithelia markers, including *Myo7a*, *Atoh1*, *Sox2*, and *Bmp4*.

(D1) scRNA-seq of the utricle at P2.

(D2) Comparison of BMP4 expression levels in integrated cell subpopulations at E14 and P2.

(E-e1) Immunofluorescent staining of BMP4 protein (indicated by yellow arrowheads) in ENHCs.

(F-f1) Post-injury, the expression of BMP4 protein in ENHCs increased.

(­G-g1) Sectional views of BMP4 staining in the cytoplasm of ENHCs.

Data are presented as the mean ± S.E.M. Scale bars: 100 μm in E; 20 μm in E1 and G. *Abbr*: E14: embryonic day 14; P2: postnatal day 2; Con: control; Gent: gentamicin; HC: hair cell; ENHC: epithelia non-hair cell; CVGC: Cochleovestibular ganglion cell; MesC: mesenchymal cell; MarC: marginal cell; SEC: sensory epithelial cell; NSEC: nonsensory epithelial cell; GC: glial cell; HBN: hindrain neuron; EndC: endothelial cell.

**Figure S3: Inhibition of BMP4 renders the functions of Notch ineffective.**

(A) Schematic diagram of experimental procedure: Post-injury, utricles from P2 *Notch1*^flox/flox^; *Sox9*-CreER*^T2^*; *ROSA26^tdTomato^*(TM) mice were split into different treatment groups. EdU was added to label proliferating cells.

(B-b2) Large numbers of Myo7a^+^ cells and *Sox9*TM^+^/EdU^+^ cells were observed in the striola in the *Notch1* knockout utricle.

(C-c4) Treatment with LDN-193189 led to a decrease in the number of regenerated HCs and *Sox*9TM^+^/EdU^+^ cells in the striola in the *Notch1* knockout utricle.

(D) Quantification and comparison of the numbers of Myo7a^+^/*Sox9*TM^+^ cells and *Sox9*TM^+^/EdU^+^ cells between the Gent and Gent+LDN groups.

Data are presented as the mean ± S.E.M. Two-way ANOVA. * *p* < 0.05, ****p* < 0.001. Scale bars: 100 μm in B and E; 20 μm in b1 and e1. *Abbr:* P: postnatal day; Tam: tamoxifen; Gent: gentamicin; LDN: LDN-193189; HC: hair cell; ENHC: epithelial non-hair cell; ES: extrastriolar; TM: tdTomato.

**Figure S4: AP-1/c-Fos acts as a critical mediator through which BMP4 promotes ENHCs reprogramming and sensitizes them to the Notch and Wnt signaling pathways.**

(A) Gentamicin control of utricle sample.
(B) Treatment with T-5224 in gentamicin damaged utricles.

(C-D) Treatment with T-5224 reduced the number of Myo7a^+^ HCs and Sox2^+^/EdU^+^ cells compared to the Gent+BMP4 group. (d3) Expression of c-Fos decreases upon the addition of T-5224, a selective c-Fos inhibitor.

(E-H) T-5224 treatment demonstrated a robust inhibitory effect, significantly decreasing the numbers of Myo7a^+^cells and Sox2⁺/EdU⁺ cells compared to either the Gent+BMP4+BIO or Gent+BMP4+DAPT groups.

(I-J) Quantification and comparison of the cell numbers.

(K) Relative mRNA expression of the AP-1 transcription factor family after BMP4 activation or simultaneous BMP4 signaling activation and c-Fos inhibition.

Data are presented as the mean ± S.E.M. Statistical analyses were performed with an unpaired t-test (for panels H and I) and a two-way ANOVA (for panels G and J). * *p* < 0.05, ** *p* < 0.01, *** *p* < 0.001, **** *p* < 0.0001. Scale bars: 100 μm in A, B; 20 μm in a1. *Abbr*: Gent: gentamicin; Pvalb: parvalbumin.

**Figure S5: IGV visualization of *Smad1* and downstream target genes *Id1, Id2, and Id3*.**

(A-B) IGV (Integrative Genomics Viewer) screenshots corresponding to the same genomic regions and experimental conditions shown in Figure 4B.

(C) Enlarged IGV views highlighting the genomic loci and read coverage for Smad1, Id1, Id2, and Id3 as presented in panel B.

**Figure S6: c-Fos binds to targets involved in reprogramming when BMP4 treatment is combined with Notch inhibition.**

(A1) Gene Ontology enrichment, culminating in a cnetplot, illustrating the predicted targets of increased c-Fos binding peaks induced by Notch inhibition along with BMP4 activation compared to the Gent+BMP4 group.

(A2) CUT&Tag differential binding peak analysis between the Gent+BMP4+DAPT group and the Gent+BMP4 group with an MA plot.

(B) Integrative Genomics Viewer screenshot showing expression peaks and enriched c-Fos binding peaks for representative regions around *Atoh1*, *Gfi1*, and *Myo6* in the utricle, revealing the potential regulatory effects of c-Fos on HC regeneration under the synergistic action of Notch inhibition and BMP4 activation.

(C) Relative expression of *Atoh1*, *Gfi1*, and *Myo6* in the Gent group, Gent+BMP4 group, and Gent+BMP4+DAPT group.

(D1-D2) Pie chart of the c-Fos CUT&Tag binding region genomic annotations in the Gent+BMP4 group and Gent+BMP4+DAPT group.

(E) Heatmap of CUT&Tag peaks in a region from 3 kb upstream from the transcription start site (TSS) to 3 kb downstream of the transcription end site (TES) in the Gent+BMP4, Gent+BMP4+DAPT, and IgG control groups.

(F) Enriched motifs identified by HOMER in the Gent+BMP4+DAPT group.

Data are presented as the mean ± S.E.M. Two-way ANOVA, * *p* < 0.05, **** *p* < 0.0001. *Abbr:* Gent: gentamicin; TSS: transcription start site; TES: transcription end site.

**Figure S7: BMP4-like small molecules enhance cell reprogramming in the neonatal mouse utricle.**

(A) Schematic diagram of experimental procedure: Utricles from P2 neonatal mice were isolated, cultured, and damaged before the addition of reagents or small molecules.

(B-b4) Control group after injury.

(C-c4) After isoliquiritigenin (ISO) treatment, a slight increase in Myo7a^+^ cells was observed in both the striolar and extrastriolar (ES) regions.

(D-d4) Treatment with 4'-hydroxychalcone (Hyd) resulted in an increase in Myo7a^+^ cells and Sox2^+^/EdU^+^ cells in the striola.

(E-e4) Simultaneous addition of DAPT and Hyd significantly increased the number of Myo7a^+^ cells and Sox2^+^/EdU^+^ cells in both the striola and extrastriola.

(F1-F2) Statistical analysis comparing the numbers of Myo7a^+^ cells and Sox2^+^/EdU^+^ cells in the striola and extrastriola in the three groups. Data are presented as the mean ± S.E.M. Two-way ANOVA. * *p* < 0.05, ** *p* < 0.01, **** *p* < 0.0001.

(G1-G2) Comparison of the number of Myo7a^+^ cells and Sox2^+^ cells in both the striola and extrastriola among the DAPT, DAPT+Hyd, and DAPT+BMP4 groups. Data are presented as the mean ± S.E.M. Two-way ANOVA. *****p* < 0.0001.

(H-h2) Control group after injury.

(I-i2) Treatment with Wnt3a resulted in a slight increase in Sox2^+^/EdU^+^ cells in the striola.

(J-j2) The addition of BIO led to a significant increase in the number of Myo7a^+^ cells and Sox2^+^/EdU^+^ cells in the striola, and the number of Myo7a+ cells increased cocurrently.

(K-k2) When CHIR was added to the culture medium, the number of Sox2^+^/EdU^+^ cells increased even more significantly.

(L1-L2) Quantification and comparison of the number of Myo7a^+^ cells and Sox2^+^/EdU^+^ cells in the striola. ***p* < 0.01, *****p* < 0.0001. Scale bars: 100 μm in B and H; 20 μm in b1 and h1.

*Abbr:* P: postnatal day; Gent: gentamicin; HC: hair cell; ENHC: epithelia non-hair cell; ES: extrastriolar; CHIR: CHIR-99021; Hyd: 4'-hydroxychalcone; ISO: isoliquiritigenin.

Figure S8: **Timeline of vestibular functional recovery following treatment with cocktail A or B.**

(A) Weekly measurements of vestibulo-ocular reflex (VOR) gain during the 4 weeks after treatment.
(B) Weekly measurements of off-vertical axis rotation (oVAR) gain over the same 4‑week post‑treatment period.
