# Supplementary material for: BMP4 cocktail promotes utricular progenitor reprogramming and vestibular functional recovery in adult mice
Source: J Adv Res. 2025 Nov 29;87:523–38. doi: 10.1016/j.jare.2025.11.065 (PMC13539158; doi:10.1016/j.jare.2025.11.065)
Supplement: Supplementary file 2 [file mmc2.docx]

**Supplementary Table1: Genotyping primers**

| CreER transgene forward | GCGGTCTGGCAGTAA AAACTATC |
| --- | --- |
| CreER transgene reverse | GTGAAACAGCATTGCTGTCACTT |
| CreER internal forward | CTAGGCCACAGAATTGAAAGATCT |
| CreER internal reverse | GTAGGTGGAAATTCTAGCATCATCC |
| tomato WT forward | AAGGGAGCTGCAGTGGAGTA |
| tomato WT reverse | CCGAAAATCTGTGGGAAGTC |
| tomato mutant reverse | GGCATTAAAGCAGCGTATCC |
| tomato mutant forward | CTGTTCCTGTACGGCATGG |
| EGFP forward | TGAAGCTTTTGGCTTTGAG |
| EGFP reverse | CCGCTGCCAAATTCTTTGG |
| *Notch1* Mutant forward | TGCCCTTTCCTTAAAAGTGG |
| *Notch1* Mutant reverse | GCCTACTCCGACACCCAATA |
| *Pou4f3*^DTR^ Forward | GTCAAAAAAGTGCCTTAGAG |
| *Pou4f3*^DTR^ Forward Mutant Reverse | CCGACGGCAGCAGCTTCATGGTC |
| *Pou4f3*^DTR^ WT Forward | CACTTGGAGCGCGGAGAGCTAG |
