# Supplementary material for: BMP4 cocktail promotes utricular progenitor reprogramming and vestibular functional recovery in adult mice
Source: J Adv Res. 2025 Nov 29;87:523–38. doi: 10.1016/j.jare.2025.11.065 (PMC13539158; doi:10.1016/j.jare.2025.11.065)
Supplement: Supplementary file 3 [file mmc3.docx]

**Supplementary Table 2. Primer pairs for qPCR.**

| **Gene** | **Forward primer** | **Reverse primer** |
| --- | --- | --- |
| *Myo6* | CCACAATGTCAAAGTTCGGTACA | GGCATCGTCCCAAGAGATTTTC |
| *Atoh1* | GGGGTTGTAGTGGACGAGC | CGTTGTTGAAGGACGGGATAAC |
| *Gfi1* | AGAAGGCGCACAGCTATCAC | GGCTCCATTTTCGACTCGC |
| *Fos* | TACTACCATTCCCCAGCCGA | GCTGTCACCGTGGGGATAAA |
| *Fosb* | ACCTGTCTTCGGTGGACTCCTT | TGGCTGGTTGTGATTGCGGTGA |
| *Junb* | AGGCAGCTACTTTTCGGGTC | TTGCTGTTGGGGACGATCAA |
| *Jun* | CCTTCTACGACGATGCCCTC | GGTTCAAGGTCATGCTCTGTTT |
| *Jund* | GAAACGCCCTTCTATGGCGA | CAGCGCGTCTTTCTTCAGC |
| *Id1* | GAACGGCGAGATCAGTGC | ATCGTCGGCTGGAACACAT |
| *Id2* | GAACACGGACATCAGCATCC | TCGACATAAGCTCAGAAGGGA |
| *Id3* | CCGCATCTCCCGATCCAG | GTCAGTGGCAAAAGCTCCTC |
| *Myc* | TCCGGGGAGGGAATTTTTGT | GCATCGTCGTGGCTGTCTG |
| *Klf4* | GGAGAAGACACTGCGTCCAG | AGAGAGTTCCTCACGCCAAC |
| *Sox2* | GCGGAGTGGAAACTTTTGTC | CTCCGGGAAGCGTGTACTTA |
| *Oct4 (Pou5f1)* | AGAGGATCACCTTGGGGTACA | CGAAGCGACAGATGGTGGTC |
