# Supplementary material for: BMP4 cocktail promotes utricular progenitor reprogramming and vestibular functional recovery in adult mice
Source: J Adv Res. 2025 Nov 29;87:523–38. doi: 10.1016/j.jare.2025.11.065 (PMC13539158; doi:10.1016/j.jare.2025.11.065)
Supplement: Supplementary file 4 [file mmc4.docx]

**Supplementary Table 3: Small molecules and chemicals.**

| **Reagent** | **Concentration** | **Vendor** |
| --- | --- | --- |
| BMP4 | 10 ng/ml | R&D Systems (Cat#314-BP) |
| T-5224 | 10 μM | Med Chem Express (Cat#HY12270) |
| LDN193189 | 1 μM | Med Chem Express (Cat# HY-12071) |
| ISO (isoliquiritigenin) | 15 μM | Med Chem Express (Cat# HY-N0102) |
| **Hyd (4-Hydroxychalcone)** | 10 μM | Sigma-Aldrich (Cat# S350664) |
| EdU (5-ethynyl-2’-deoxyuridine) | 10 μM | Life Technologies (Cat# C10340) |
| 4-OHT (4-Hydroxytamoxifen) | 5 nM | Med Chem Express (Cat# HY-16950B) |
| IDPN (3,3'-Iminodipropionitrile) | 5 μl/g | TCI Shanghai (Cat# TCI-I0010) |
| Tamoxifen | 0.20 mg/g | Sigma-Aldrich (Cat# T5648-1G) |
| P407 | 10 μl | Sigma-Aldrich (Cat# 16758) |
| CHIR-99021 | 3 μM | Med Chem Express (Cat# HY-10182) |
| BIO | 5 μM | Sigma-Aldrich (Cat# 361550) |
| Wnt3a | 20 ng/ml | R&D Systems (Cat# 5036-WN) |
| Gentamicin sulfate | 2 mM | Sigma-Aldrich (Cat# G1914) |
| DAPT | 50 μM | Med Chem Express (Cat# 208255-80-5) |
| LY-411575 | 5 μM | Med Chem Express (Cat# HY-50752) |
| Neomycin trisulfate salt hydrate | 1 mM | Sigma-Aldrich (Cat# N6386) |
| Diphtheria Toxin | 25 ng/g | List Biological Laboratories（Cat #150） |
