# Supplementary material for: BMP4 cocktail promotes utricular progenitor reprogramming and vestibular functional recovery in adult mice
Source: J Adv Res. 2025 Nov 29;87:523–38. doi: 10.1016/j.jare.2025.11.065 (PMC13539158; doi:10.1016/j.jare.2025.11.065)
Supplement: Supplementary file 5 [file mmc5.docx]

**Supplementary Table 4: List of antibodies information.**

| **Antibody** | **Source** | **Cat; RRID; Application** | **Dilution** |
| --- | --- | --- | --- |
| DAPI | Thermo Fisher Scientific | Cat# D1306; AB_2629482; IF | 1:800 (IF) |
| Phalloidin | Thermo Fisher Scientific | Cat# A22287; AB_2620155; IF | 1:200 (IF) |
| Mouse anti-BMP4 | Abcam | Cat# ab124715; AB_10974254**;** IF/WB | 1:200 (IF) |
| Rabbit anti-Myo7a | Proteus Biosciences | Cat# 25-6790; AB_10015251; IF/WB | 1:800 (IF) |
| Mouse IgG1 anti-Parvalbumin | Sigma-Aldrich | Cat# P3088; AB_477329; IF | 1:500 (IF) |
| Goat anti-Sox2 | Santa Cruz Biotechnology | Cat# sc-17320; AB_2286684; IF/WB | 1:300 (IF) |
| Rat/IgG2a, kappa anti-Sox2 | Thermo Fisher Scientific | Cat# 14-9811-82; AB_11219471; IF/WB | 1:300 (IF) |
| Rabbit anti-c-Fos | Cell Signaling Technology | Cat# 2250; AB_2247211; IF/WB/ChIP | 1:50 (CUT&Tag)  1:100 (IF)  1:1000 (WB) |
| Unconjugated Rabbit IgG | Millipore | Cat# 12-370; AB_145841; IP/WB | 1:100 (CUT&Tag) |
| Mouse IgG1 anti-Ki-67 | Cell Signaling Technology | Cat# 9449; AB_2715512; IF/WB | 1:1000 (IF) |
| Goat polyclonal anti-phospho-Smad1/5/8 | Santa Cruz Biotechnology | Cat# sc-12353; AB_656614; IF/WB | 1:200 (IF) |
| Mouse IgG1 anti-GAPDH | Thermo Fisher Scientific | Cat# MA1-16757; AB_568547; WB | 1:1000 (WB) |
| Alexa Fluor™ Donkey anti-Goat IgG 488 | Thermo Fisher Scientific | Cat# A11055; AB_2534102 | 1:500 (IF) |
| Alexa Fluor™ Donkey anti-Goat IgG 555 | Thermo Fisher Scientific | Cat# A21432; AB_2535853 | 1:500 (IF) |
| Alexa Fluor™ Donkey anti-Mouse IgG 488 | Thermo Fisher Scientific | Cat# A21202; AB_141607 | 1:500 (IF) |
| Alexa Fluor™ Donkey anti-Mouse IgG 555 | Thermo Fisher Scientific | Cat# A31570; AB_2536180 | 1:500 (IF) |
| Alexa Fluor™ Donkey anti-Mouse IgG 647 | Thermo Fisher Scientific | Cat# A31571; AB_162542 | 1:500 (IF) |
| Alexa Fluor™ Donkey anti-Rabbit IgG 488 | Thermo Fisher Scientific | Cat# A21206; AB_2535792 | 1:500 (IF) |
| Alexa Fluor™ Donkey anti-Rabbit IgG 555 | Thermo Fisher Scientific | Cat# A31572; AB_162543 | 1:500 (IF) |
| Alexa Fluor™ Donkey anti-Rabbit IgG 647 | Thermo Fisher Scientific | Cat# A31573; AB_2536183 | 1:500 (IF) |
| Goat Anti-Rabbit IgG-HRP | Abmart | Cat# M21002; AB_2713951 | 1:5000 (WB) |
| Goat Anti-Mouse IgG HRP | Abmart | Cat# M21001; AB_2713950 | 1:5000 (WB) |
| Goat polyclonal anti-phospho-Smad1 /5/8 | Santa Cruz  Biotechnology | Cat#sc-12353;  AB_656614 | 1:300 (CO-IP) |
| Normal Rabbit IgG Control | Millipore | Cat #12-370;  AB_145841 | 1:300 (CO-IP) |
