# Supplementary material for: BMP4 cocktail promotes utricular progenitor reprogramming and vestibular functional recovery in adult mice
Source: J Adv Res. 2025 Nov 29;87:523–38. doi: 10.1016/j.jare.2025.11.065 (PMC13539158; doi:10.1016/j.jare.2025.11.065)
Supplement: Supplementary file 6 [file mmc6.docx]

Supplementary Table5: Abbreviation in Figures.

| ***Abbr*** | Full name | ***Abbr*** | Full name |
| --- | --- | --- | --- |
| E14 | embryonic day 14 | *E*16 | embryonic day 16 |
| P | postnatal day | P2 | postnatal day 2 |
| 6 h | 6 hours | Con | control |
| Gent | gentamicin | Neo | neomycin |
| LDN | LDN193189 | CHIR | CHIR-99021 |
| Hyd | 4'-hydroxychalcone | ISO | isoliquiritigenin |
| 4-OHT | 4-hydroxytamoxifen | Tam | tamoxifen |
| HC | hair cell | SC | supporting cell |
| ENHC | epithelia non-hair cell | S | striola |
| ES | extrastriola | Pvalb | parvalbumin |
| TM | tdTomato | N.S | no significant |
| TSS | transcription start site | TES | transcription end site |
| CVGC | Cochleovestibular ganglion cell | MesC | mesenchymal cell |
| MarC | marginal cell | SEC | sensory epithelial cell |
| NSEC | Non-sensory epithelial cell | GC | glial cell |
| HBN | hindrain neuron | EndC | endothelial cell |
| EC | effective concentration | con | concentration |
| IC | inhibitory concentration | DT | Diphtheria Toxin |
| RF | Right front limb | RH | Right hind limb |
| LF | Left front limb | LH | Left hind limb |
| VOR | Vestibulo-ocular reflexes | VSR | Vestibulo-spinal reflex |
| aVOR | angular vestibular-ocular reflex | LY | LY411575 |
