# Supplementary material for: BMP4 cocktail promotes utricular progenitor reprogramming and vestibular functional recovery in adult mice
Source: J Adv Res. 2025 Nov 29;87:523–38. doi: 10.1016/j.jare.2025.11.065 (PMC13539158; doi:10.1016/j.jare.2025.11.065)
Supplement: Supplementary file 7 [file mmc7.docx]

**Table S6. Summary of the efficacy of the Hyd/ISO cocktail on vestibular function recovery in a mouse model of vestibular damage.**

| **Function Assessment**  **Category** | **Control Group**  (Mean ± SEM, n) | **Cocktail A Group**  (Mean ± SEM, n) | **p-value**  **(VS control)** | **Cocktail B Group**  (Mean ± SEM, n) | **p-value**  **(VS control)** |
| --- | --- | --- | --- | --- | --- |
| HC counts(S) | 24.00±1.08 (n = 4) | 92.50±2.47 (n = 4) | < 0.0001 | 61.00±3.70 (n = 4) | < 0.0001 |
| HC counts (ES) | 61.00±5.98 (n = 4) | 82.25±3.20 (n = 4) | 0.0020 | 71.00±3.01 (n = 4) | 0.16 |
| VOR Gain (4 week) | 0.120±0.035 (n = 4) | 0.30±0.06 (n = 4) | 0.0001 | 0.21±0.04 (n = 4) | 0.021 |
| OVAR gain (4 week) | 0.03±0.06 (n = 3) | 0.19±0.04 (n = 3) | 0.0002 | 0.13±0.01 (n = 3) | 0.021 |
| oVEMP **(**Amplitude**)** | 2.46±0.306 mV (n = 5) | 5.19±0.20 mV (n = 5) | 0.0001 | 2.81±0.22 mV (n = 6) | 0.58 |
| Gait Metrics | / | Improvement of front and hind limbs | | Improvement of front limbs | |
